# Comprehensive analysis of transcriptome profiles in hepatocellular carcinoma

**DOI:** 10.1186/s12967-019-2025-x

**Published:** 2019-08-20

**Authors:** Yu Jin, Wai Yeow Lee, Soo Ting Toh, Chandana Tennakoon, Han Chong Toh, Pierce Kah-Hoe Chow, Alexander Y.-F. Chung, Samuel S. Chong, London L.-P.-J. Ooi, Wing-Kin Sung, Caroline G.-L. Lee

**Affiliations:** 1 Department of Biochemistry, Yong Loo Lin School of Medicine, National University of Singapore, Singapore, 119077 Singapore; 2 Genome Institute of Singapore, Singapore, Singapore; 3 Division of Medical Sciences, Humphrey Oei Institute of Cancer Research, National Cancer Centre Singapore, Level 6, Lab 5, 11 Hospital Drive, Singapore, 169610 Singapore; 4 Duke-NUS Medical School, Singapore, 169547 Singapore; 5 Department of Surgery, Singapore General Hospital, Singapore, 169608 Singapore; 6 Department of Paediatrics, Yong Loo Lin School of Medicine, National University of Singapore, Singapore, 119228 Singapore; 7 Department of Laboratory Medicine, National University Hospital, Singapore, 119074 Singapore; 8 Department of Surgical Oncology, National Cancer Centre Singapore, Singapore, 169610 Singapore; 9 School of Computing, National University of Singapore, Singapore, Singapore

**Keywords:** Liver cancer, HBV integration, Chimeric transcripts, Differentially expressed genes, Somatic mutations

## Abstract

**Background:**

Hepatocellular carcinoma is the second most deadly cancer with late presentation and limited treatment options, highlighting an urgent need to better understand HCC to facilitate the identification of early-stage biomarkers and uncover therapeutic targets for the development of novel therapies for HCC.

**Methods:**

Deep transcriptome sequencing of tumor and paired non-tumor liver tissues was performed to comprehensively evaluate the profiles of both the host and HBV transcripts in HCC patients. Differential gene expression patterns and the dys-regulated genes associated with clinical outcomes were analyzed. Somatic mutations were identified from the sequencing data and the deleterious mutations were predicted. Lastly, human-HBV chimeric transcripts were identified, and their distribution, potential function and expression association were analyzed.

**Results:**

Expression profiling identified the significantly upregulated TP73 as a nodal molecule modulating expression of apoptotic genes. Approximately 2.5% of dysregulated genes significantly correlated with HCC clinical characteristics. Of the 110 identified genes, those involved in post-translational modification, cell division and/or transcriptional regulation were upregulated, while those involved in redox reactions were downregulated in tumors of patients with poor prognosis. Mutation signature analysis identified that somatic mutations in HCC tumors were mainly non-synonymous, frequently affecting genes in the micro-environment and cancer pathways. Recurrent mutations occur mainly in ribosomal genes. The most frequently mutated genes were generally associated with a poorer clinical prognosis. Lastly, transcriptome sequencing suggest that HBV replication in the tumors of HCC patients is rare. HBV-human fusion transcripts are a common observation, with favored HBV and host insertion sites being the HBx C-terminus and gene introns (in tumors) and introns/intergenic-regions (in non-tumors), respectively. HBV-fused genes in tumors were mainly involved in RNA binding while those in non-tumors tissues varied widely. These observations suggest that while HBV may integrate randomly during chronic infection, selective expression of functional chimeric transcripts may occur during tumorigenesis.

**Conclusions:**

Transcriptome sequencing of HCC patients reveals key cancer molecules and clinically relevant pathways deregulated/mutated in HCC patients and suggests that while HBV may integrate randomly during chronic infection, selective expression of functional chimeric transcripts likely occur during the process of tumorigenesis.

## Background

Hepatocellular carcinoma (HCC) is the 6th most common and the 2nd most fatal cancer worldwide [1]. The dismal prognosis is primarily due to its late presentation and limited therapeutic options [2], highlighting an urgent need to better understand HCC to identify biomarkers capable of detecting early-stage HCC, as well as uncover therapeutic targets to develop novel therapies for HCC.

Gene expression profiling of HCC patients have led to the identification of several gene signatures associated with clinical characteristics as well as deregulated molecular pathways [3, 4]. Genes involved in cell cycle progression, DNA repair, cytoskeletal and extracellular matrix were frequently reported to be up-regulated in HCC while immune response and metabolic enzyme genes were found to be down-regulated [5].

In addition to de-regulation of gene expression, mutation signatures had also been characterized in HCC. Using next generation sequencing, somatic mutations in TERT promoter, TP53 and CTNNB1 were frequently reported to be mutated in HCC patients [6]. Somatic mutations were enriched in the promoter of the TERT gene and occurred in > 50% HCC patients, while protein-altering mutations were frequently observed in TP53 and CTNNB1 genes [7]. Wnt signaling, telomere maintenance and cell cycle control were significantly altered by mutations in HCC [6], while chromatin remodelers e.g. ARID1A, ARID2 and BRD7 were also found to be mutated and de-regulated in HCC patients [6, 8].

One of the most commonly associated etiological factor for HCC in East Asia is chronic hepatitis B virus (HBV) infection. Approximately 5% of the world’s population (350–400 million people) is affected by HBV [9], with persistent HBV infection leading to chronic liver disease and accounting for ~ 50% of all HCC cases.

Hepatitis B virus has a ~ 3.2 kb genome containing four overlapping reading frames responsible for the production of seven viral proteins: S (encoding the large, middle and small surface proteins), P (encoding the viral polymerase), C (encoding the antigens “e” and core protein) and X (encoding the regulatory HBx protein). HBV replicates via an RNA intermediate, the pre-genomic RNA (pgRNA), which is 3.5 kb long.

Hepatitis B virus is thought to play a key role in HCC development by integrating its genome into the host genome. HBV genome integration events are observed in a high proportion of HBV-related HCC patients as reported by several studies using high-throughput sequencing [10–13]. Although HBV seems to integrate randomly into the host genome, these studies revealed several genes are recurrently targeted by the viral integration events including the *TERT*, *MLL4* and *FN1*, suggesting that HBV integration events may have functional consequences on the host.

Interestingly, we and several other groups [10–12, 14, 15] also observed that the region between nucleotide 1600–1900 within the viral genome which corresponds to the 3′-end of the *HBx* gene and the 5′-end of the *Precore* gene is significantly involved in insertion into the host genome. Our previous study has also identified the same region as being preferentially involved in the structural alteration within the viral genome, especially deletion and inversion events [11].

Although the integration sites in the host genome and structural alterations within the HBV have been well characterized at the genomic level, the status of HBV transcripts have not been comprehensively analyzed [16]. One study that performed transcriptome sequencing of HBV-positive cell lines discovered an HBx-LINE1 chimeric transcript, which was reported to be expressed in 21 of 90 (23%) HCC patients [17]. This chimeric transcript was correlated with poorer patient survival and may function as a long non-coding RNA to promote HCC development [17], highlighting the importance of comprehensively characterizing the transcriptome of tumour and adjacent non-tumor tissues in HCC patients. A recent study characterized HBV integration through RNA sequencing and reported preferential sites of integration in the proximity of telomeres [12]. Several other studies characterized the transcriptome profile of HCC patients, mainly analyzing the host transcripts in only a few (≤ 10) HCC patients, but not the viral transcripts [18–20].

In this study, we performed whole transcriptome sequencing of 25 pairs of HCC tumor and adjacent non-tumor samples to comprehensively evaluate the profile of both the host and HBV transcripts in HCC patients.

## Methods

### Tissue samples

**Fig. 1:**
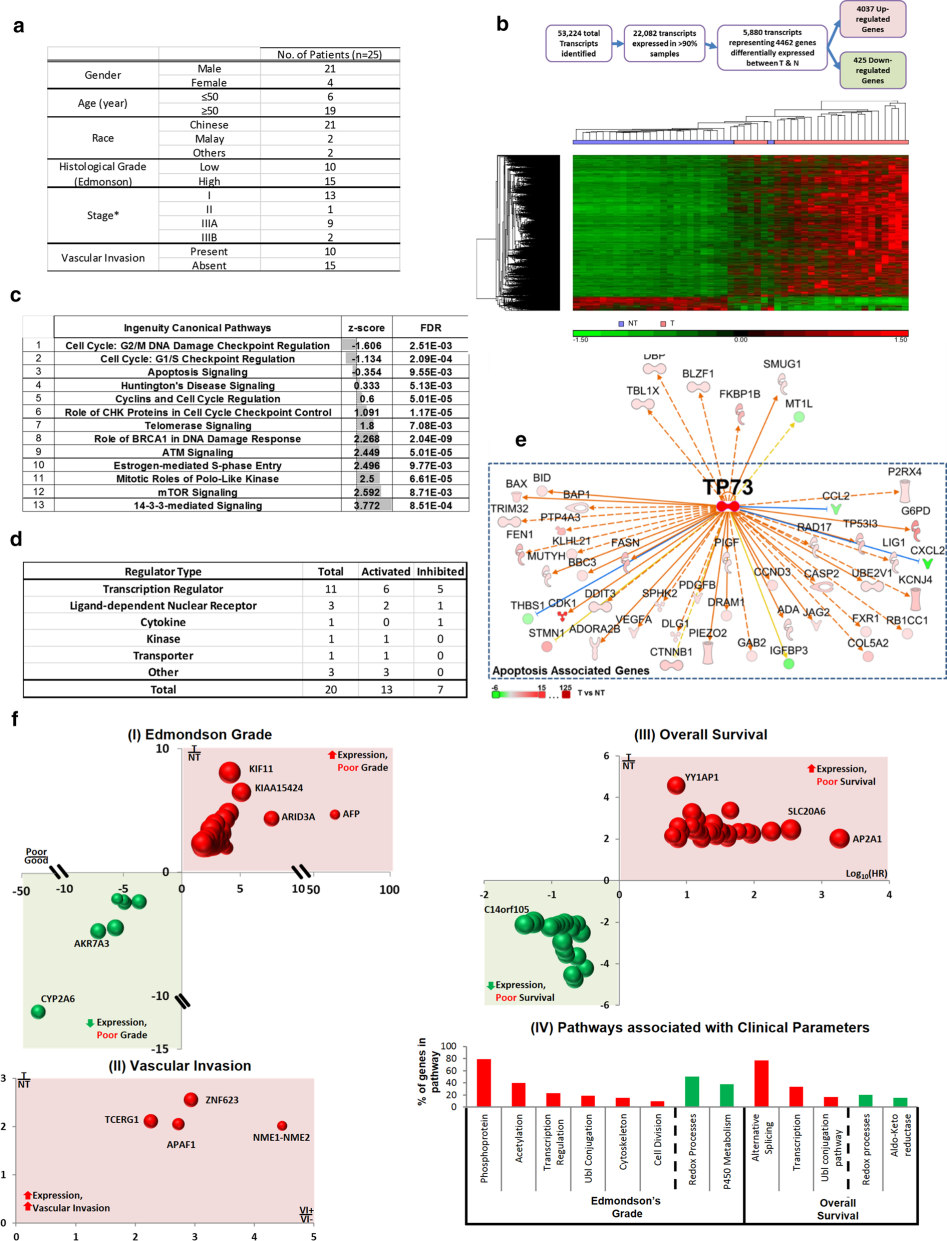
Differentially expressed genes between tumors and adjacent non tumor tissues. **a** Demographic and clinicopathological data of the 25 HCC patients recruited in this study. HCC staging was classified according to tumor-node-metastasis system by the American Joint Committee on Cancer. **b** Differentially expressed genes between tumor and adjacent non-tumor tissues. Top: The number of differentially expressed transcripts/genes at various stages of the workflow. Bottom: Heat-map of the 4462 differentially expressed genes in tumors and adjacent non-tumor patient samples. **c** Significant pathways associated with the differentially expressed genes between tumors and adjacent non-tumors. FDR < 0.01. Z-score predicts the activation status of the pathway. FDR denotes False Discovery Rate. **d** Characteristics of upstream regulators. **e** Network of genes associated with the most activated regulatory molecule, TP73. **f** Differentially expressed genes associated with clinical parameters. I–III The genes associated with clinical parameters. The X-axis represents the fold-change of gene expression between tumors and adjacent non-tumorous tissues (T/N). The Y-axis represents the fold-change of gene expression between patients with less favorable clinical outcome (i.e. higher grade (poor), greater vascular invasion (VI+) and poorer survival) versus those with more favorable clinical outcome (i.e. lower grade (good), less vascular invasion (VI−) and better survival). The size of the bubble represents the − log-_2_ (FDR) of gene expression between T and NT. HR denotes hazard ratio. (IV) Biological pathways associated with differentially expressed genes associated with the various clinical characteristics. The X-axis shows the pathways that are significantly associated with clinically associated genes for the different clinical phenotype. A red bar shows genes which are upregulated in tumors relative to the non-tumors associated with a worse clinical outcome, while a green bar represents genes downregulated in tumors relative to the non-tumors associated with a worse clinical phenotype

Twenty-four pairs of HBV-positive and one pair of HBV-negative HCC and adjacent non-tumor samples were selected for whole transcriptome sequencing in this study. Adjacent non-tumor (NT) tissues were taken from the resected specimen at the furthest margin away from the tumor. All the tissues were collected anonymously from the National Cancer Centre Tissue Repository with informed consent from the patients and prior approval from the NCCS Institutional Review Board (NCC_IRB_No_2007/437/B). Fifty-six percent of the patients are stage 1 or 2 HCC patients and 44% are stage 3 (A&B) patients. Demographic and clinical-pathological data of the patients can be found in Fig. 1a.

### Transcriptome analysis for chimeric transcripts

Total RNA was isolated from the 25 pairs of HCC and adjacent non-tumor tissues using RNeasy Mini Kit (Qiagen). Briefly, the tissues were homogenized in buffer RLT (Qiagen) containing 1% β-mercaptoethanol using the gentleMACS dissociator (Miltenyi Biotec). Total RNA was isolated using the RNeasy Mini Kit (Qiagen) according to the manufacturer’s instructions. The quality assessment was performed using a Nanodrop ND-1000 Spectrophotometer (NanoDrop Technologies) and Agilent 2100 Bioanalyzer (Agilent). The mRNA was first enriched using a polyA sequence. The mRNA was subsequently fragmented to 130–160 nucleotides. Random hexamers were then used to reverse transcribe the RNA into cDNA. Sequencing adaptors were then ligated using the Illumina TruSeq RNA Sample Prep Kit v2. Fragments of ~ 150-bp long were selected by gel electrophoresis and amplified by 13 cycles of PCR. High-throughput paired end (PE-90) sequencing providing 5 Gigabases per sample was performed on the Illumina HiSeq™ 2000 platform, as described in the Illumina mRNA expression analysis protocol (http://www.illumina.com). Whole transcriptome sequencing data was deposited in Gene Expression Omnibus with series entry GSE105130.

RNA-seq libraries were aligned to a combined genome consisting of the hg19 genome and HBV genome sequences using the Tophat aligner. From the resulting alignments, chimeric reads that contain both human and HBV sequences were identified and the exact breakpoints were determined, as described in Additional file 1: Methods.

The breakpoints of the chimeric transcripts were then mapped to different genic regions including promoters (5 kb upstream transcription starts site), exons (including 5′- and 3′-UTRs), introns and non-coding RNA. The number of the breakpoints between nucleotide 1600–1900 in the HBV genome was also determined. We then evaluated whether there was enrichment of fusion sites in the different genic regions of the human genome using a random sampling approach, as described in Additional file 1: Methods.

### Profiling differential expression of host genes/transcripts

Transcript and individual exon expression was estimated from Tophat output BAM files based on hg19, Refseq version 2015.02.02 annotation using Partek Genomic Suite 6.6. Reads per kilo-base per million mapped reads (RPKM) were calculated for each transcript and exon. Paired sample t-tests, followed by Benjamini–Hochberg correction, were performed and genes showing more than a twofold change and FDR < 0.05 were identified as differentially expressed genes. Pathway analyses of the differentially expressed genes were performed using Ingenuity Pathway Analysis, as described in Additional file 1: Methods.

### Analysis of somatic mutations in tumor tissues

The somatic mutations were identified using the alignment files from Tophat output and the steps are described in Additional file 1: Methods. Tumor-specific mutations were annotated in the reference genome (hg19) using snpEff [21]. The functional effects of missense mutations were predicted using Polyphen-2 [22]/SIFT [23], and mutations that were predicted to be damaging by both programs were determined to be deleterious. The nonsense mutations that are more than 50 bp from the downstream exon boundary and not in the last exon were determined to cause nonsense-mediated decay (NMD) [24]. We further investigated the host gene expression and identified the NMD-causing mutations that correspond to more than a 1.5-fold change in the same tumor tissue as potentially functional and expression-associated mutations.

### Clinical association and survival analysis

The association between somatic mutations or human gene expression with clinical parameters (Fig. 1a) was analyzed in this study using the R Project for Statistical Computing. Cox proportional hazards tests were performed to identify whether gene expression is associated with overall survival outcome, and Kaplan–Meier survival analyses were employed to determine if the presence of genetic mutations is significantly associated with survival outcome. All the statistical tests for different types of clinical parameters and survival are described in Additional file 1: Methods.

## Results

### HCC patient transcriptome profiles reveal that differentially expressed transcripts are mainly involved in cell cycle regulation

Transcriptome sequencing was performed on the tumors and adjacent non-tumorous tissues from 25 mainly male, Chinese HCC patients (Fig. 1a). A total of 53,224 transcripts were identified from deep transcriptome sequencing, and 22,082 transcripts were expressed in > 90% of the samples examined (Fig. 1b top). Details of the deep transcriptome sequencing are provided in Additional file 1: Methods. A total of 5879 transcripts representing 4462 genes showed significantly differential expression between the T and NT (fold change (FC) > 2; FDR < 0.05) and hierarchical clustering of these genes was generally able to appropriately classify most of the tumor tissues from non-tumor tissues, except for the misclassification of 5 tumor tissues which were clustered with ‘non-tumor tissues (Fig. 1b bottom). All the five tumors that clustered with non-tumor tissues exhibited low Edmondson grade (grades 1 and 2) [25], which may account for their expression signatures being more similar to the non-tumor tissues. Of the 4462 differentially expressed genes, 4037 genes were found to be significantly up-regulated, while only 425 genes were down-regulated in the tumor tissues compared with the adjacent non-tumor tissues. Ingenuity^®^ Pathway Analysis (IPA) identified 13 canonical pathways that were significantly enriched (FDR < 0.01) with these differentially expressed genes (Fig. 1c). Nearly half of the canonical pathways (6/13) are associated with changes in cell cycle regulation, while others are associated with DNA damage response, cell-survival and apoptosis signaling (Fig. 1c). Six of these pathways including ATM, mTOR and 14-3-3 signaling have positive Z-scores above 2, suggesting that these pathways are likely to be activated in HCC (Fig. 1c). Nineteen of these differentially expressed genes were predicted to play a role in 5 or more pathways, with CDK1 being associated with the most number (7) of pathways (Additional file 1: Figure S1A). Notably, the expression of CDK1 in late-stage (Stages 3A and 3B) tumors is significantly higher (p-value < 0.05, Student’s t-test) than in early-stage (Stages 1 and 2) tumors (Additional file 1: Figure S1B).

A total of 20 upstream regulators were found to be significantly associated (Z-score > 2 or < − 2; FDR of overlap < 0.01) with the 4462 differentially expressed genes (Additional file 1: Figure S2). As evident in Fig. 1d (and Additional file 1: Figure S2), majority of the upstream regulators are activating, and belong to the family of transcription regulators and ligand-dependent nuclear receptors. These upstream regulators primarily modulate target genes in the cell-cycle (including CDK1), apoptosis, chromosome/DNA pathways (Additional file 1: Figure S2). Notably, the upstream regulator with the highest activation Z-score (5.6), TP73 is itself significantly up-regulated in the tumors of HCC patients (Additional file 1: Figure S2) and is predicted to activate the target genes mainly in the pathway of apoptosis regulation (Fig. 1e). Hence, dysregulation of TP73 and apoptosis may play key roles in HCC development of these patients and TP73 could be a useful biomarker.

### Differentially expressed genes are associated with clinical parameters

Out of the 4462 differentially expressed genes, 110 genes were found to be differentially expressed and are associated with clinical characteristics in HCC that indicate poorer prognosis (Fig. 1f, Additional file 1: Table S1). Fifty-six genes were associated with advanced Edmondson grades, four were associated with the presence of vascular invasion, and 45 with poorer overall survival. In addition, five genes (NUP133, SKP2, TBL1X, AKR7A3 and SHMT1) were associated with both advanced Edmondson grades and poorer overall survival. Of the 61 genes associated with poorly differentiated tumors (Edmondson grades 3 and 4) and poorer prognosis, 53 genes are up-regulated and eight are down-regulated in the tumors of HCC patients. The up-regulated genes primarily function in transcriptional regulation, post-translational modifications (acetylation, conjugation and protein phosphorylation) and cell division (Fig. 1f (IV)). On the other hand, four of the eight down-regulated genes associated with higher tumor grade are involved in oxidation–reduction, while three are involved in P450 metabolism (Fig. 1f (IV)). Of the 50 genes associated with poorer overall survival, 30 were significantly up-regulated in the tumors of HCC patients. Nearly 80% of these up-regulated genes have alternatively spliced forms and 33% were involved in transcription (Fig. 1f_IV), including POLR2H, YY1AP1, ZNF552, WDR5, UCHL5, ADNP, ZNF765, TBL1X, ZNF585A and ERCC2. Five of the genes were involved in ubiquitin-like protein conjugation (Fig. 1f_IV). Amongst the other 20 down-regulated genes associated with poorer overall survival, four genes were involved in oxidation–reduction processes, of which, three were aldo–keto reductases.

Hence, several differentially expressed genes are associated with clinical characteristics, with most of the up-regulated genes (84/110, 76%) associated with poorer prognosis (i.e. higher Edmondson grade, vascular invasion and/or poorer survival), having roles in post-translational modifications, cell-cycle and/or transcriptional regulation. The 26 down-regulated genes associated with higher Edmondson grade and/or poorer survival is primarily involved in oxidation–reduction modulating and oxidative stress/damage management.

### Majority of somatic mutations are within coding regions with potential to affect function

**Fig. 2:**
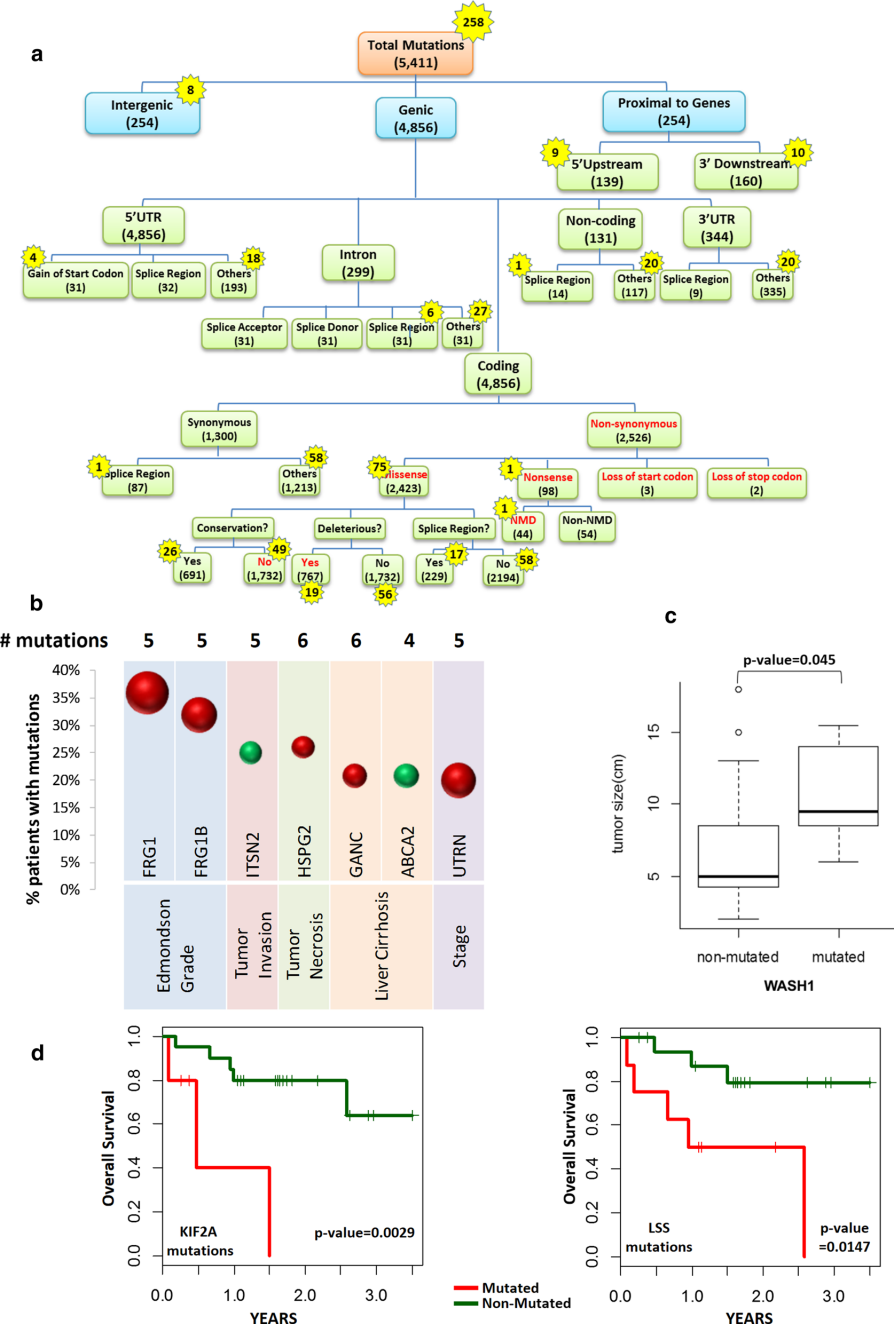
Tumor-specific somatic mutations identified from RNA-seq. **a** Distribution of tumor-specific somatic mutations in different genomic regions. The number of total somatic mutations in various genomic regions and the numbers of recurrent mutations (numbers in yellow stars). Most of the somatic mutations reside in genic regions, and missense mutations in coding sequences accounted for ~ 50% of genic mutations. 5′ Upstream: mutation occurring within 5 kb upstream of genes. 3′ Downsteram: mutation occurring within 5 kb downstream genes. Splice donor: mutation that changes one of 2 bases at the 5′ end of an intron. Splice acceptor: mutation that changes one of 2 bases at the 3′ end of an intron. Splice region: mutation within 1–3 bases of the exon or 3–8 bases of the intron flanking the intron–exon boundary. Deleterious: mutation predicted to be damaging to protein function by both Polyphen-2 and SIFT algorithms. NMD: a mutation predicted to cause nonsense-mediated decay. **b** Association of mutations in HCC patients with clinical characteristics. The percentage of patients with mutations (Y-axis) for the various genes (X-axis) associated with the various clinical phenotype (X-axis below the genes). Red balls denote bad prognosis (e.g. associated with high Edmondson grade tumor, late stage, necrosis or liver cirrhosis) while green balls represents good prognosis (protective genes associated with no tumor invasion/liver cirrhosis). Size represents significance of association i.e. larger size, smaller p-value. **c** Association of mutations in HCC patients with tumor size. Box plot show tumor size of patients with and without mutation in WASH1 gene. **d** Mutations in Genes associated with overall survival. Patients with mutations in KIF21A (left panel) and LSS (right panel) correspond to significantly shorter survival time (p-value < 0.01, Kaplan–Meier test). Green lines represent patients with no mutations in the gene while red lines represent patients with mutations in the gene

The transcriptome of HCC patients was further investigated to identify tumor-specific somatic mutations that may play important roles in tumorigenesis. A total of 5411 tumor-specific mutations were identified with each patient having an average of 230 somatic mutations (Additional file 1: Figure S3A). Figure 2a shows the distribution of the somatic mutations in the different genomic regions and their predicted functionality. Notably, ~ 90% (4856) of the somatic mutations are genic, of which ~ 79% (3826) are within coding regions, with 66% (2526) of coding mutations being non-synonymous. A majority (> 95%, 2423) of non-synonymous mutations are missense mutations, while 3.9% (98) are nonsense mutations (gain of stop codon). Moreover, three and two mutations result in the loss of start and stop codons, respectively (Fig. 2a).

Approximately 70% (1732) of the missense mutations resulted in non-conservative amino acid changes, while ~ 30% (767) were predicted to be deleterious by both Polyphen-2 and SIFT algorithms (Fig. 2a). Other mutations predicted to be deleterious include mutations that affect start/stop codons, or splice donor or acceptor sites (Fig. 2a, Additional file 1: Figure S3B). These deleterious mutations reside in 787 genes, which are primarily involved in ATP-binding, ATPase and helicase activity, as well as GTPase-mediated signal transduction (Additional file 1: Figure S3C).

Nearly 45% (44) of the nonsense mutations were predicted to result in nonsense-mediated decay (NMD) (Fig. 2a). Nonsense mutations in seven different genes, each in seven HCC patients, were predicted to cause NMD and were associated with > 1.5 fold decrease in expression in the tumor tissues compared with the adjacent non-tumor tissues (Additional file 1: Figure S4). Notably, in three of the seven patients that carried the NMD mutations in CHD1L, DPF2 and BRD7, the genes were down-regulated in these tumor tissues while the same genes in the other patients showed significant up-regulation (FDR < 0.05) (Additional file 1: Figure S4A–C). Analyses of HCC patient data from The Cancer Genome Atlas (TCGA) cohort also revealed that one TCGA patient had the same nonsense mutation in the BRD7 gene, similar to one of the patients in this study. The expression of this BRD7 gene in this TCGA patient was found to be also associated with 5.74 decrease in gene expression. Hence, nonsense mutations may play a role in modulating gene expression in HCC tumors.

Greater than 95% (5153) of these somatic mutations were unique (non-recurrent), while 258 (5%) mutations were found in 2–6 patients (Fig. 2a, yellow stars). Likewise, ~ 90% (231) recurrent mutations were genic, of which ~ 58% (135) genic mutations resided within coding region of genes and ~ 56% (75) coding mutations were non-synonymous. Missense mutations constituted ~ 99% of all non-synonymous recurrent mutations, with only one nonsense mutation that was predicted to cause NMD. A high percentage (~ 65%) of recurrent missense mutations resulted in non-conservative changes, while ~ 25% were predicted to be deleterious. These recurrent mutations resided within 194 genes, which were significantly over-represented in the ribosome and involved in translation (Additional file 1: Figure S5). The most common recurrent somatic mutation found in 6 (24%) tumors was the synonymous Cys159Cys within the FRG1 gene, which was reported to interact with the mRNA sequence independently and was postulated to regulate pre-mRNA splicing [26, 27].

Genes harboring the 4856 genic mutations were found to reside in 193 pathways, with 27 pathways harbouring genes that were mutated in ≥ 80% (≥ 20/25) of the HCC patients, and 23 pathways carrying genes with recurrent mutations (Additional file 1: Figure S6). As expected, cancer pathways contained the most number of mutated genes (71) harboring the most number of mutations (106), with every patient carrying at least one mutation in one of the genes of a cancer pathway. Phosphatidylinositol signaling, focal adhesion, endocytosis, ubiquitin-mediated proteolysis and extracellular matrix (ECM)-receptor pathways were also found to be significantly enriched (FDR < 0.05) with mutated genes.

Taken together, tumor-specific mutations in HCC patients reside in important gene regions and are likely to affect gene function. Different pathways including those modulating the microenvironment were enriched with mutated genes. Ribosomal genes involved in translation were most enriched with genes having recurrent mutations (Additional file 1: Figure S5).

### Frequently mutated genes are associated with clinical characteristics

Mutations in several genes, which occurred in at least 20% (5/25) of HCC patients, were found to be significantly associated with different clinical parameters (Fig. 2b). Occurring in > 30% of HCC patients, mutations within the FRG1 and FRG1B genes may affect the regulation of pre-mRNA splicing [26, 27], and was found to be significantly associated with tumors of higher histological grade (p-value = 0.003 and 0.008). Mutations of UTRN gene occurred in 20% of the patients and was significantly associated with late-stage HCC development (p-value = 0.009) (Fig. 2b). Mutations of GANC gene occurred in 21% patients and were found to be associated with liver cirrhosis (p-value = 0.037). Moreover, HSPG2 mutations, occurring in 26% of HCC patients, showed significant association with liver necrosis (p-value = 0.048) (Fig. 2b). Mutations in WASH1, an actin nucleation-promoting factor, found in 20% of patients, were associated with larger tumor size (Fig. 2c). Significantly, mutations in KIF21A, which encodes mitotic kinesin protein, and LSS, which encodes lanosterol synthase, were found to be associated with a significant decrease in overall survival (KIF21A: p-value = 0.0029 and LSS: p-value = 0.0147) (Fig. 2d). On the other hand, mutations in ITSN2 were found in tumors without vascular invasion, while mutations of ABCA2 gene were only identified in the tumors of patients without liver cirrhosis (Fig. 2b).

### Pre-genomic RNA within HCC patients is mainly incomplete

**Fig. 3:**
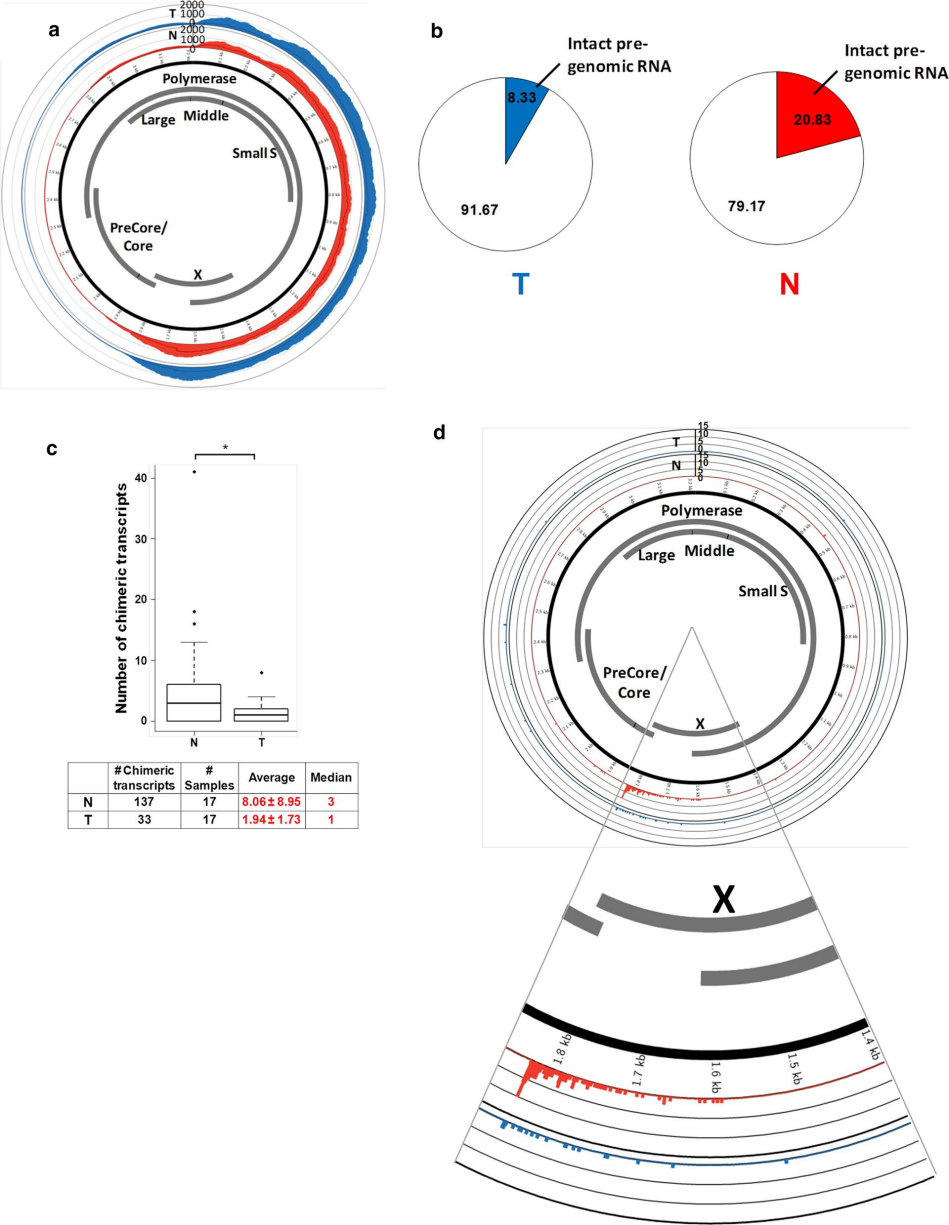
Coverage of HBV genome and proportion of patients with potentially intact pre-genomic RNA. **a** Coverage of HBV genome by sequencing reads. The circos plot shows the average coverage of each nucleotide of HBV genome by the sequencing reads. Red and blue histograms show the average coverage in the 24 N and T, respectively. Coverage is significantly higher in the Pre-S and X genes. **b** Proportion of patients with potentially intact pre-genomic RNA. As the pre-genomic RNA is 3.5 kb and covers the entire HBV genome, the pre-genomic RNA is considered incomplete if any region of the HBV is not detected. Intact pre-genomic RNA is likely to be found in 20.83% and 8.33% of N and T samples, respectively, suggesting that HBV replication is not a common event in HCC patient liver. **c** Less variety of chimeric transcripts in the tumor of HCC patients. Boxplot showing average and median number of different chimeric transcripts. The table shows the total number of different chimeric transcripts, number of samples which contain chimeric transcripts, the average and median number of chimeric transcripts detected in T and N samples. The number of different chimeric transcripts detected in T is significantly lower in compared to the N samples (p-value < 0.05, paired t-test) suggesting that a subset of functional chimeric transcripts is selected in the process of tumorigenesis. **d** Circos plot showing the distribution of fusion sites on HBV genome. The fusion sites between the HBV and host sequences in the chimeric transcripts are significantly located in the region between 1600 and 1900 of the HBV genome (near the end of the HBx gene) in both non-tumor and tumor samples (p-value < 0.001, random sampling test)

Transcriptome sequencing also provides us with the opportunity to examine HBV RNA in HCC patients. As HBV replicates via an RNA intermediate, the presence of this RNA intermediate (pgRNA) in HCC samples serves as an indication of viral replication. pgRNA is 3.5 kb in length and has to be kept intact to preserve the entire viral genome during viral replication. Figure 3a shows that the transcriptome coverage for different HBV gene regions by sequencing reads are highly variable. Majority of the HBV transcripts map to the Pre-S and X gene. Unlike the Pre-S and X genes, significantly less reads were observed for the precore–core, large surface antigen, and the 5′ end of the polymerase compared with other HBV genes, suggesting that pgRNA is present at low levels in these patients since polymerase and pre-core are expressed from the pgRNA. As shown in Fig. 3b, only 20.83% and 8.33% of non-tumor and tumor samples, respectively, have potentially intact pgRNA, as defined by complete coverage of the HBV genome with sequencing reads. Hence, HBV replication is likely a rare event in HCC patients’ liver, especially in the tumors, as intact pgRNA is rarely observed.

### Fewer chimeric transcripts were observed in tumor compared with non-tumor samples

As HBV genome integration events are often observed in HCC samples [11], it is important to examine whether these integrated viral sequences are expressed and whether the human sequences adjacent to the insertion sites are expressed along with the integrated viral sequences to form chimeric transcripts.

A total of 33 and 137 unique chimeric transcripts were observed in 17 tumor (T) and 17 non-tumor (N) samples respectively, giving an average of 1.94 and 8.06 chimeric transcripts in their respective T and N samples (Fig. 3c). The median numbers of chimeric transcripts were 1 and 3 in the T and N samples respectively (Fig. 3c). Statistical analysis revealed that significantly more chimeric transcripts were observed in the N compared with the T samples (p-value < 0.05, paired t-test).

### HBV sequences inserted via the end of HBx are expressed

Consistent with a previous report [11] which showed that the 3′-end of HBx and 5′-end of precore/core (nucleotides 1600–1900 of HBV genome) are significantly involved in HBV integration events, the fusion points between HBV and human sequences in the chimeric transcripts observed in this study were also located primarily in the same region (p-value < 0.001, random sampling test) (Fig. 3d). Hence, integration of viral sequences occurring at the 3′ end of the HBx gene (within the 1600–1900 nucleotide region) into host DNA results in the expression of chimeric transcripts containing primarily 5′ end of HBx and human sequences at the 3′ end of the HBx gene.

Notably, the transcript coverage of the HBV genome immediately after the favored fusion sites decreased significantly (Fig. 3d). This suggests that HBV integration events disrupt the viral genome leading to incomplete pgRNA, which likely renders the virus replication-defective.

### HBV-host chimeric transcripts are primarily the fusion of the HBx gene and repetitive elements within introns of human genes

**Fig. 4:**
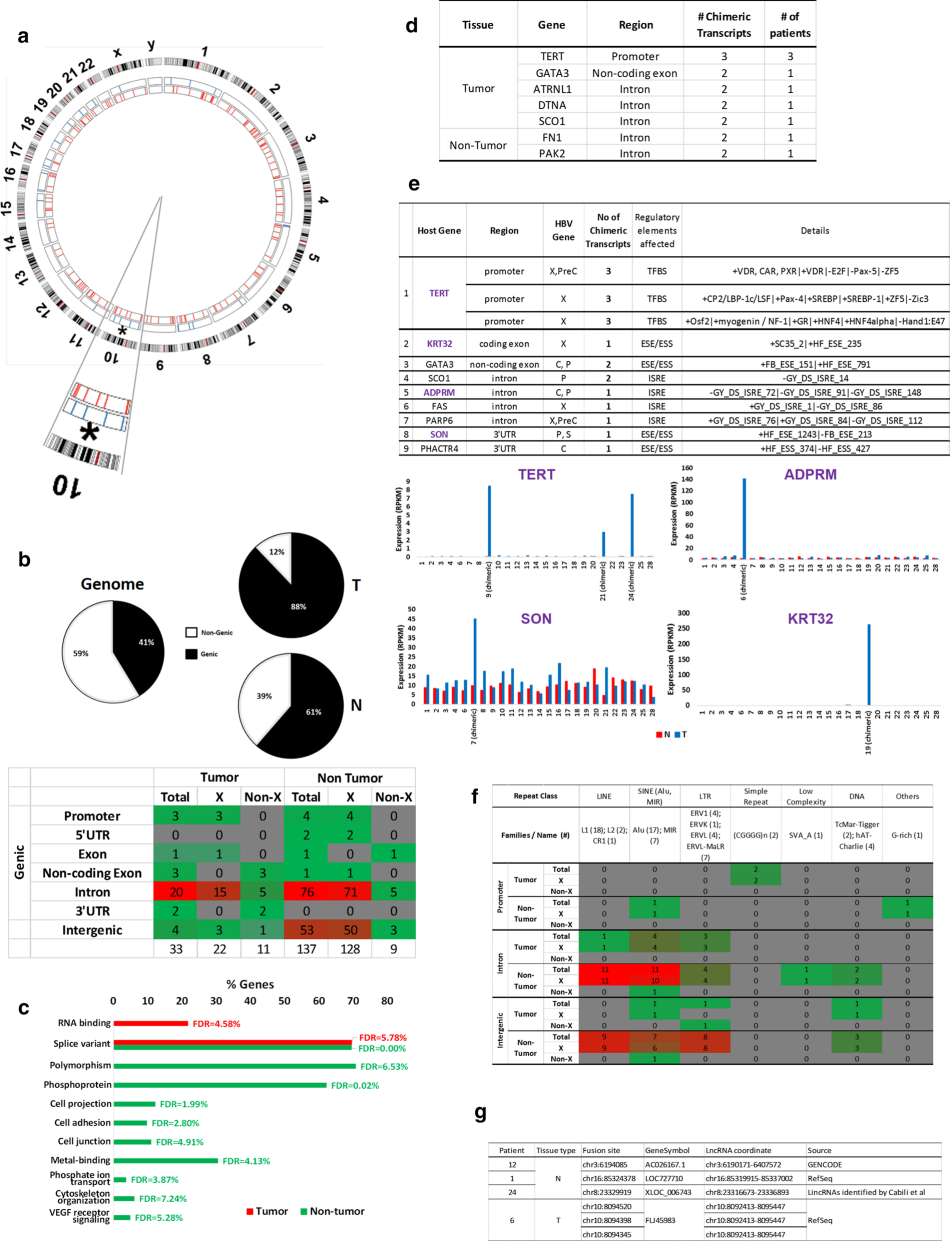
Distribution of fusion points on chromosomes. **a** Circos plot showing the distribution of fusion points on human chromosomes (hg19). Each red or blue bar represents a fusion site on the corresponding chromosome in N and T samples, respectively. **b** Proportion of fusion sites in genic and intergenic regions. Top panel: The left pie chart shows the proportion of genic and intergenic region in human genome. The two pie charts on the right show the proportion of HBV-host chimeric transcripts from the genic and intergenic regions in T and N samples, respectively. Genic region includes promoters, 5′- and 3′-UTRs, coding or non-coding exons and introns while intergenic region excludes the genic region. Bottom panel: Distribution of fusion points on functional regions of genes. **c** Functional annotation of genes with viral integration sites in N and T. Red bars represent the functional annotation of genes with viral integration sites identified in the tumor tissues while the green bars correspond to the functional annotation of genes with integration sites in non-tumor tissues. **d** Table showing genes fused with HBV in at least two chimeric transcripts. **e** Tumor chimeric transcripts predicted to alter regulatory elements and their association with expression. Top panel: Table showing putative regulatory sites of genes that are predicted to be affected by viral integration in tumor tissues. Genes with fusion sites that are associated with expression changes are in bold purple. Bottom panel: association between tumor chimeric transcripts and host gene or adjacent exon expression. Red bars represent gene/exon expression in non-tumor while blue bars represent expression in tumor tissues. **f** Distribution of fusion points in different classes of repeat regions. **g** Distribution of fusion points in long non-coding RNAs

We have previously reported that HBV preferentially integrates into chromosome 10 in tumor cells; this integration was correlated with poorly differentiated tumors [11]. Consistent with our previous observations [11], we also found that RNA mapping to chromosome 10 was significantly enriched as the fusion partner of HBV to form chimeric transcripts (Fig. 4a).

Building upon our previous observation that genic regions are enriched with HBV integration events (50% in tumors and 43.9% in non-tumors) [11], we found that HBV-human chimeric transcripts were even more significantly enriched with human sequences from genic regions, especially in tumors (88%) (Fig. 4b top), suggesting selection pressure for human genic sequences to be incorporated into the HBV-human chimeric transcripts in tumors (p-value < 0.001, Fisher’s exact test). Majority of the chimeric transcripts observed are between HBx and introns or intergenic regions, especially in non-tumor tissue (Fig. 4b bottom). Notably, only HBx, but not the other HBV transcripts, was observed to be fused to the regulatory region of genes including the promoter and 5′ untranslated region (UTR) (Fig. 4b bottom).

In HCC patients, HBV-human chimeric transcripts were observed to be fused to different regions of 105 different human genes, with tumor chimeric transcripts fused to various regions of 23 different genes, while adjacent non-tumorous liver chimeric transcripts fused to various regions of 82 different genes (Additional file 1: Table S2). The 23 genes identified in chimeric transcripts from HCC tumor tissues were primarily involved in RNA binding and enriched for alternative splice variants (Fig. 4c, red bars). Similarly, the 82 genes with HBV integration sites identified in non-tumor tissues were enriched for alternative splice variants, as well as polymorphic proteins and phosphoproteins. Moreover, they are over-represented in cell projection, cell junction, cell adhesion regulation, and are involved in phosphate transport, signaling pathways and cytoskeleton organization (Fig. 4c, green bars).

In tumor tissue, the promoter, non-coding exons or introns of five genes were found to be fused to HBV in 2 or more chimeric transcripts, with three different regions of the TERT promoter being fused to HBV in chimeric transcripts from three different patients (Fig. 4d). In adjacent non-tumorous liver tissue, the introns of two genes were observed to be fused to two different chimeric transcripts (Fig. 4d).

The junctions where HBV fuse with human genes were predicted to alter various regulatory elements, including transcription factor binding sites (TFBS), exon splice enhancers/silencers (ESE/ESS) and intron splice regulatory elements (ISRE) in 11 chimeric transcripts (Fig. 4e). Notably, the expression of 4 of these genes (TERT, ADPRM, SON and KRT32) were significantly higher in tumor tissues expressing chimeric transcripts compared to tissues without the fusion transcripts (Fig. 4e, bottom bar graphs). This suggests that the fusion of viral sequences in these genes may alter transcription factor binding or splicing regulation leading to increased expression of the gene/exon.

As HBx-LINE1 fusion transcripts were previously reported to be tumor promoting and correlated with poorer patient survival [17], we evaluated whether the chimeric transcripts were fused to repeat elements. HBV was fused to various classes of repeat sequences of the human genome in ~ 40% (71/170) of the chimeric transcripts. Notably, except for 3 fusion transcripts with non-HBx as the viral partner, HBx is the dominant viral gene that was fused to human repeat sequences in the other 68 chimeric transcripts (Fig. 4f). LINE and SINE were the dominant family of repeat sequences found in the chimeric transcripts (Fig. 4f). Although 18 chimeric transcripts contained LINE1 as previously reported, only one chimeric transcript in the tumor tissue was found to carry the LINE1 repeat (Fig. 4f). The LINE1 repeat in this chimeric transcript mapped to the intron of a zinc-finger ZC3H3 gene and was fused to the HBx gene.

Six chimeric transcripts were found to overlap with long non-coding RNAs (Fig. 4g). Three fusion sites identified in three different non-tumor tissues were found to be located in AC026167.1, LOC727710 and XLOC_006743, respectively. The other three sites were all identified in the tumor tissue of patient 6 and may fuse with FLJ45983.

## Discussion

Deep transcriptome sequencing of HCC patients provides three important types of information about these patients, namely the profiles of their mRNA expression and transcript mutations, as well as the existence and characteristics of HBV-Human chimeric transcripts.

mRNA expression profiles of these patients revealed significant up-regulation of 4037 genes and significant down-regulation of 425 genes. These dysregulated genes are primarily associated with cell-cycle regulation, DNA damage response, cell-survival and apoptosis signaling. Significantly, we identified TP73, which is significantly up-regulated in the tumors of HCC patients as the most significant up-stream regulator, activating target genes mainly in the apoptosis regulation pathway. This is consistent with previous observations of up-regulation of TP73 in HCC [28, 29] and its well-known role in apoptosis regulation [30–33]. Although the expression of TP73 was not significantly associated with any clinical characteristics in this study, likely due to limited sample size, higher TP73 was reported to be significantly associated with lower mean survival in a larger cohort of 193 HCC patients [34] and higher levels of lymph node metastasis, vascular invasion and pathological staging in breast cancer [35]. In light of this, TP73 could thus potentially serve as a useful biomarker and promising target for therapy.

Differential expression of 110 genes in the tumors of HCC patients were associated with Edmondson grade, vascular invasion and/or overall survival. Similar observations were made with the transcriptome sequencing data of HCC (LIHC) patients from TCGA (Additional file 1: Table S3). Approximately 90% of genes that were significantly associated with Edmondson grade, in this study, was also found to be significantly associated with Edmondson grade, in 366 LIHC patients from TCGA (Additional file 1: Table S3). Similarly, > 75% and all of the genes significantly associated with overall survival and vascular invasion, respectively, in this study also showed consistent trends in the TCGA cohort (Additional file 1: Table S3). Hence, clinical association identified in this study was highly concordant with data of HCC patients from TCGA.

Higher expression of up-regulated genes in HCC tumors that are primarily involved in post-translational modification, cell-division and/or transcriptional regulation is associated with higher Edmondson grade and worse overall survival. On the contrary, lower expression of down-regulated genes in HCC tumors and primarily involved in oxidation–reduction is associated with worse prognoses of poorly differentiated tumors and worse overall survival. Hence, it would be worthwhile to further characterize these genes in these pathways for their role in modulating prognosis of these patients.

From transcriptome sequencing, mutation profiles revealed that tumor-specific mutations in HCC patients tended to reside in important gene regions, likely to affect their function. An average of 149–291 somatic mutations were observed for each patient which is similar to a previous report which identified 2–445 mutations per patient using whole-exome sequencing of East Asian HCC patients [36] but higher than the 94–101 coding variants per patient identified through transcriptome sequencing of only three HCC patients in yet another report [37]. Hence, greater than 5000 mutations, primarily non-synonymous missense mutations in the coding region of genes, were found in the tumors of these patients. Nearly 100 nonsense mutations were observed and 44 were predicted to result in nonsense-mediated mRNA decay (NMD). Interestingly, in three patients with NMD mutations in three different genes (CHD1L, DPF2 and BRD7), the specific genes were down-regulated in the tumors of these patients but significantly up-regulated in all other patients. The same NMD mutation in the BRD7 gene was also found in a patient from TCGA and BRD7 gene expression in that tumor was also found to be decreased by 5.74 fold, highlighting that nonsense mutations may play an important role in regulating gene expression.

Genes carrying somatic mutations are significantly enriched in various pathways including those modulating the tumor microenvironment, e.g. extracellular matrix (ECM)-receptor pathways. Five percent of somatic mutations observed are recurrent. These recurrent mutations are primarily missense mutations and genes with recurrent mutations are primarily involved in the ribosome or translation. Several frequently mutated genes were found to be associated mainly with worse prognosis with higher tumor grades (FRG1, FRG1B), later stage (UTRN), tumor necrosis (HSPG2), liver cirrhosis (GANC), larger tumor size (WASH1) and worse overall survival (KIF21A and LSS), although mutations in ABCA2 and ITSN2 were associated with better prognosis with no liver cirrhosis and no tumor invasion, respectively. Evaluating for mutations in these genes in HCC patients may be useful as prognostic biomarkers.

Transcriptome sequencing of HCC patients can also provide useful insights about the characteristics of the HBV virus in these HCC patients. HBV is frequently reported to be integrated into the genome of HCC samples [10, 11, 13–15]. Although HBV has been well-established as a strong risk factor for HCC, the virus has not been comprehensively characterized at the transcript level in HCC patients. It has also been debatable whether HBV is replicating in the liver of HCC patients. Since HBV replicates via an RNA (pgRNA) intermediate, a complete pgRNA is essential for the replication of HBV. HBV replication in the tumor or adjacent non-tumorous liver tissues was thus evaluated by assessing the presence of the complete pgRNA. Few intact pgRNAs are observed in majority of the patients (Fig. 3a, b), suggesting that HBV replication is rare, especially in the tumors of HCC patients.

As HBV integration events are commonly observed in HCC samples, it is important to understand the functional consequences of these integration events. Although the integration sites have been well-characterized by several groups including ours [10, 11, 13–15], the virus transcripts and especially the virus-host chimeric transcripts have not been systematically characterized until recently.

In this study, 33 and 137 unique HBV-Human chimeric transcripts were found in 17 tumor and 17 non-tumor tissues respectively, suggesting that there were significantly more HBV-host chimeric transcripts in non-tumor compared to tumor tissue. This is consistent with the observations of a previous study characterizing HCC transcriptome of 22 HCC patients, which reported more HBV-human fusions (161) in non-tumorous tissues compared to matched HCC tissues (33 fusions) [38]. Notably, most of the chimeric transcripts in the tumors were found to fuse with genic sequences, which is even more significant than what we observed at the genomic level [11]. In 40% of chimeric transcripts, HBV was fused to repeat sequences especially the LINE and SINE family of repeats. Similar to our previous observations [11], we also observed that sequences in chromosome 10 were significantly enriched as the fusion partner of HBV in the tumors.

Interestingly, a recent study, which also performed DNA sequencing of 426 HCC paired samples after HBV enrichment followed by validation of 12 tumor samples using RNA sequencing, reported preferential integration of HBV in chromosome 17 with higher HBV integration frequency in tumors [12], which is contrary to our observations. One possible reason for this discrepancy could be due to the differences in the techniques used for characterizing HBV chimeric transcripts. The above-mentioned study performed sequencing only after enrichment with HBV capture probes [12], which may bias the types of chimeric transcripts that are identified, and may not effectively detect transcripts where only a short region of HBV is fused to human sequence. On the other hand, our study performed deep sequencing of the entire transcriptome of every patient and thus should be able to identify all types of transcripts including transcripts with shorter regions of HBV fused to human sequences. Other possible reasons for the discrepancy could be due to differences in the patient cohort or the genotype of HBV examined, and further studies are needed to clarify this.

Favored sites of integration within HBV remains at the 3′ end of the HBx gene, as previously observed at genomic level [11]. This region overlaps with direct repeat region DR1, which is involved in HBV replication [39]. It is thus consistent with our observation that pre-genomic RNAs were rarely detected in both tumor and non-tumor tissues (Fig. 3b), since integration in the vicinity of DR1 (nucleotides 1600–1900) of the HBV genome observed in this (Fig. 3d) and previous genomic study [11] would disrupt HBV replication.

Favored fusion sites within the host are primarily the introns in tumor tissue and introns and intergenic regions in non-tumorous tissues. Various regions within 23 and 82 host genes are fused to HBV in the tumor and non-tumor tissues respectively. These 105 genes are enriched for alternative splice variants, with genes fused to HBV in tumors primarily involved in RNA binding, while genes fused to HBV in the non-tumor tissues enriched in polymorphic proteins, phosphoprotein, cytoskeleton and involved in phosphate transport and VEGF receptor signaling. In seven genes (TERT, FN1, ATRNL1, DTNA, GATA3, PAK2 and SCO1), more than one HBV-human chimeric transcripts were identified. The most favored/frequently observed site of integration is the promoter of the TERT gene, which was found to be fused to HBV in three different HCC patients. Similar observations of HBV integration mainly into the promoter of TERT have also been previously reported by at least six other groups including our own (Additional file 1: Figure S7) [10, 11, 14, 17, 40]. Twenty-five other host genes fused to HBV in this study were also reported to be fused to HBV in at least one other study (Additional file 1: Figure S7), with 1 (FN1) reported to be fused to HBV in four other studies and two (ATRNL1 and CPS1) reported to be fused to HBV in three other studies [10–13]. A previous report interrogating the transcriptome in 44 tumors and 4 non-tumor tissues of HBV^+^ HCC patients found that MLL4 was recurrently fused with HBV [41]. However, our group did not observe integration into MLL4 gene [11] nor MLL4-HBV fusion transcripts (this study). One possible reason could be due to the differences in the HBV genotypes in the different studies, with HBV-C genotype being predominant in Dong et al’s [41] study, while most of our HCC patients carry the HBV-B genotype.

Taken together, these observations suggest that in HCC patients, while HBV integrates randomly in the genome during a chronic infection, there seems to be a selection of functional chimeric transcripts during the process of tumorigenesis accounting for the significantly less varied chimeric transcripts and favored sites of fusion in the tumors compared with non-tumorous tissues.

## Conclusions

In summary, transcriptome sequencing of HCC patients revealed TP73 as nodal molecules regulating apoptotic genes. One hundred and ten genes were found to be significantly associated with clinical outcomes including Edmondson grade, vascular invasion and/or survival. Signatures of somatic mutations demonstrated that they are mainly non-synonymous, and affect genes in the microenvironment cancer pathways. Lastly, HBV-human chimeric transcripts were enriched in genic regions, affecting different regulatory elements. These chimeric transcripts demonstrated favored sites of integration in tumor tissues, suggesting possible selection of functional chimeric transcripts during tumorigenesis.

## Supplementary information

**Additional file 1.** Methods, Figures S1–S8, Tables S1–S3.

## Data Availability

Whole transcriptome sequencing data generated in the current study is available in Gene Expression Omnibus with series entry GSE105130. The datasets are currently not publicly available but are available from the corresponding author on reasonable request.

## Abbreviations

DEG: differentially expressed gene
ESE: exonic splicing enhancer
ESS: exonic splicing silencer
*FN1*: fibronectin 1
HBV: hepatitis B virus
HBx: hepatitis B virus X Protein
HCC: hepatocellular carcinoma
ISRE: intronic splicing regulatory elements
*MLL4*: myeloid/lymphoid or mixed-lineage leukemia 4
NGS: next generation sequencing
NT: non-tumor
ORF: open reading frame
PCR: polymerase chain reaction
pgRNA: pre-genomic RNA
T: tumor
*TERT*: telomerase reverse transcriptase
TF: transcription factor
TSS: transcription start site
UTR: untranslated region
