# Supplementary material for: Comprehensive analysis of transcriptome profiles in hepatocellular carcinoma
Source: J Transl Med. 2019 Aug 20;17:273. doi: 10.1186/s12967-019-2025-x (PMC6701074; doi:10.1186/s12967-019-2025-x)
Supplement: Supplementary file 1 — Additional file 1. Methods, Figures S1–S8, Tables S1–S3. [file 12967_2019_2025_MOESM1_ESM.pdf]

Comprehensive Analysis of Transcriptome Profiles in Hepatocellular Carcinoma Patients

Yu Jin[1#], Wai Yeow Lee[1#], Soo Ting Toh[1#], Chandana Tennakoon[2], Han Chong Toh[3], Pierce Kah-Hoe Chow[4,5], Alexander Y-F Chung[5], Samuel S. Chong[6,7], London L.-P- J Ooi[5,8], Wing-Kin Sung[2,9], Caroline G-L Lee[1,3,4,*]

Additional file 1

1. Methods
2. Figures S1-S8
3. Tables S1-S3

Methods

Generation of consensus sequences for HBV genome

The consensus HBV sequences of each genotype A-H were used as the reference genome. The consensus sequences were generated by aligning the HBV sequences of each genotype using Jalview. The number of HBV sequences with available genotype information and used for generation of each consensus sequences were 327, 503, 931, 684, 207, 110, 14 and 20 for genotype A, B, C, D, E, F, G and H respectively.

Mapping of sequencing reads to the genomes and fusion transcript detection

RNA-seq libraries were first aligned to human and HBV genomes using Tophat. From the resulting alignments, read pairs that had at least one read mapped to the HBV genome or with at least one side unmapped were extracted. These read pairs were aligned with Blast to a database containing known HBV strains. Next, read pairs having at least one side mapping to an HBV strain were extracted. Chimeric reads and reads that originated from HBV were presented in this final set of reads. An in-house RNA-seq read assembler PETA was used to assemble all these reads.

The pairs of reads used in the resulting assemblies were separated into three groups; 1) read pairs where both sides were used in the same assembly; 2) reads where only one side was used in some assembly while its mate was never used; and 3) read pairs where the two sides were used in two different assemblies. The third group of reads was unreliable and discarded. Read assembly was then repeated with the remaining reads.

The first group of reads, which can lead to confident assemblies, were re-assembled using PETA. The second group of reads were aligned to the human genome using BLAST, and clustered according to the best hit given by BLAST. Two reads, which mapped within 1000 bp from each

other, were added to the same cluster. Finally multiple sequence alignment was performed using an in-house program MSA to generate consensus sequences for these clusters.

The resulting assemblies and consensuses were aligned to human and HBV databases using BLAST to determine whether they are chimeric, and then to determine the exact breakpoints.

Deep transcriptome sequencing of 25 pairs of HCC and adjacent non-tumor samples generated an average of 56,547,541 clean reads and 5,089,278,683 clean bases, with an average Q20 percentage of 96.76%. About 94% of the total raw reads were successfully mapped to the reference hg19 and HBV genome. Among these mapped reads, 99.97% was mapped to the hg19, while 0.03% was mapped to HBV genome (Figure S8A).

In order to assess whether the sequencing depth is sufficient, saturation curves were generated for the 50 samples sequenced. As additional reads were sampled, the number of splice junctions detected increased very rapidly at first, and reached plateau when ~40% of the reads were sampled, suggesting that the sequencing depth was sufficient (Figure S8B).

Evaluation of preferred viral or chromosomal sites or gene region for formation of fusion transcripts

Based on the total number of fusion transcripts observed in the transcriptome sequencing, the same number of sites were randomly sampled *in silico* from the HBV or human genome. The region (nucleotide 1,600-1,900) in the HBV genome or human chromosome where these randomly sampled fusion sites reside were recorded. The process was repeated 1,000,000 times to generate an empirical distribution of fusion sites for the region of interest in the HBV genome or each human chromosome. Using chromosome 10 as an example, the observed number of fusion sites on chromosome 10 was 7. Based on 1,000,000 times of random samplings, there were 610

sampling cycles generating more than 7 fusion sites on chromosome 10. Hence the empirical p-value was 6.1×10^{-4} (610/1,000,000). After multiple test correction, FDR was found to be 0.01464, which is less than 0.05, and thus is considered statistically significant. The R Project for Statistical Computing was used to perform the random sampling and the probability calculations. Similar method was used to assess whether there was significant enrichment of breakpoints in various genic regions.

Pathway analysis of differentially expressed genes and genes with somatic mutations

Differentially Expressed Genes were analyzed using Ingenuity Pathway Analysis to identify significantly enriched Canonical Pathways and Upstream Regulators. A right-tailed Fisher's Exact Test (FET) was used to test for significant overlap between the differentially expressed genes between T and NT, as well as the genes in the categories of Canonical Pathways and Upstream Regulators respectively, as defined by Ingenuity Knowledge Base™. After multiple test correction, FDR of less than 0.01 was defined to be statistically significant for the FET.

An Activation Z-score was used to predict the activation status of the canonical pathways and upstream regulators based on the expression level of the differentially expressed genes associated with the canonical pathway and upstream regulators. The associated differentially expressed genes were compared with their expected expression level based on literature curated in Ingenuity Knowledge Base™. An absolute Z-score above 2 was considered to be significant and this allows for the prediction of the activation or inhibition status of a pathway or regulator.

To identify the functions of genes carrying somatic mutation, the Database for Annotation, Visualization and Integrated Discovery (DAVID) was utilized for the annotation of functions.

Gene ontology (biological process – FAT, cellular compartment –FAT, molecular function-FAT) and KEGG pathway analysis were performed for the gene groups of interests. After Benjamini-Hochberg correction, FDR<0.05 was used as the cutoff for statistical significance.

Detection of somatic mutations

The output BAM files from Tophat alignments were used for generating the pileup files using Samtools and the consensus of the reads covering each base were obtained. For each position where the consensus base was different from the reference (indicated by the presence of "alternative base"), statistics of the numbers of high-quality (Phred-score quality score ≥ 50) reads carrying the alternative and reference bases were computed. Only the mutation events that contain at least five high-quality alternative reads in the tumor samples and five high-quality reads with reference allele in the non-tumorous samples were determined as tumor-specific somatic mutations in this study.

Clinical association and survival analysis

Most of the clinical parameters were binary variables e.g. vascular invasion and no vascular invasion. We grouped the patients with histological grade=1 or 2 as low-grade group, while those with grade=3 or 4 as high-grade group. Patients at stage I and II were grouped as early-stage, patients while the others were at late stage.

For analysis of association between clinical parameters and gene expression, we first calculated the ratio of gene expression in tumor to expression in paired non-tumor tissue i.e. T/NT ratio and performed log transformation of all the T/NT ratios. Then we compared the log ratios in two groups (E.g. patients with and without vascular invasion for each of the human genes) using Student's t-test followed by Benjamini-Hochberg correction. Genes that exhibit significant

differences in T/NT ratios between the two groups (FDR < 0.05) were identified to be associated with clinical parameters.

Based on the presence of somatic mutations in a particular gene, we grouped the patients into two categories and built a 2×2 contingency table corresponding to a particular clinical phenotype e.g. with and without vascular invasion. FET was used to identify the association between mutations and clinical phenotype, and associations with p-value<0.05 were reported.

Cox proportional hazards modeling was employed to analyze the association between gene expression and overall survival. For each of the genes, the ratio of the gene expression in tumor to that in the adjacent non-tumor tissue was calculated for every patient and used as the explanatory variable in the Cox proportional hazards regression model.

Kaplan-Meier survival analysis was performed for identification of mutated genes associated with overall survival outcome. For each of the human genes in which mutations were identified in the tumors of at least five HCC patients, we classified the patients into two groups based on the presence of somatic mutations within the genes. Kaplan-Meier curves were plotted for the two groups and Log-rank test was used for comparison of the two survival curves. Statistical significance was determined as Log-rank p-value<0.05.

Figure S1. Differentially expressed genes predicted to be involved in ≥5 different pathways

A

	Number of Pathways	Gene	FDR	Fold Change (T/NT)	1433 pathway	mTOR Signaling	Mitotic Role of Polo-Like Kinase	Estrogen-mediated S-Phase Entry	ATM Signaling	Role of BRCA1 in DNA Damage Response	Telomerase Signaling	Role of CHK Proteins in Cell Cycle Checkpoint Control	Cyclins and Cell Cycle Regulation	Huntington's Disease Signaling	Apoptosis Signaling	Cell Cycle:G1/S Checkpoint Regulation	Cell Cycle:G2/M DNA Damage Checkpoint Regulation
1	7	**CDK1**	7.14E-05	18.4			■		■	■	■	■	■				■
2	6	**E2F1**	8.63E-04	21.3				■	■	■		■	■			■	
3	6	**PPM1L**	6.64E-04	2.4		■	■			■	■	■	■				
4	6	**PPP2R1A**	1.73E-02	2.1		■	■			■	■	■	■				
5	6	**PPP2R3B**	8.64E-05	2.4		■	■			■	■	■	■				
6	6	**PPP2R4**	2.39E-05	2.4		■	■			■	■	■	■				
7	6	**PPP2R5A**	6.50E-06	2.5		■	■			■	■	■	■				
8	6	**PPP2R5D**	1.88E-05	2.5		■	■			■	■	■	■				
9	5	**ATR**	8.24E-06	2.2					■			■	■			■	■
10	5	**CDK2**	9.76E-04	2.3				■		■		■	■			■	
11	5	**CHEK2**	5.09E-04	3.1			■		■		■	■					■
12	5	**E2F3**	2.94E-04	4.1				■		■		■	■			■	
13	5	**E2F4**	2.78E-04	2.1				■		■		■	■			■	
14	5	**E2F5**	1.06E-02	3.5				■		■		■	■			■	
15	5	**E2F6**	7.56E-03	2.4				■		■		■	■			■	
16	5	**HRAS**	1.63E-04	2.3	■	■				■				■	■		
17	5	**MAPK1**	1.29E-05	2.3	■	■				■				■	■		
18	5	**MAPK3**	1.60E-04	2.5	■	■				■				■	■		
19	5	**PRKCA**	5.33E-05	2.7	■	■				■				■	■		

B

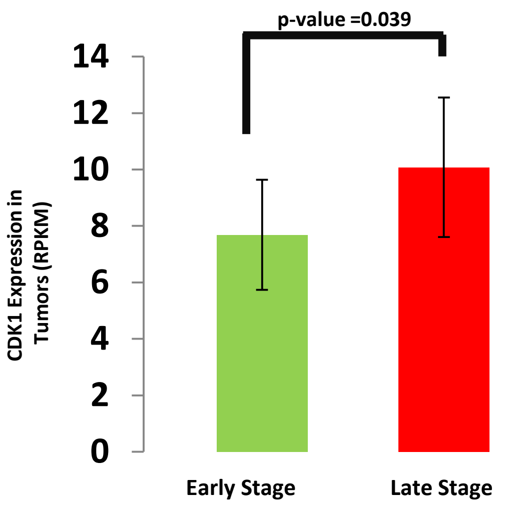

Figure S2. Upstream regulators significantly associated with differentially expressed genes.

A Summary of upstream regulators significantly associated with differentially expressed genes.

	REFSEQ	GENE NAME	FDR (T/NT)	FC (T/NT)	Molecule Type	Predicted Activation State	Activation z-score	p-value of overlap	Functional Anotation of Target Genes	Enrichment Score
1	NM_005427	**TP73**	4.36E-03	124.0	transcription regulator	Activated	5.6	1.21E-03	Regulation of Apoptosis	4.96
2	NM_021953	**FOXM1**	3.72E-04	25.0	transcription regulator	Activated	3.3	6.56E-03	cell cycle and p53 signaling	4.70
3	NM_130439	**MXI1**	2.30E-02	7.0	transcription regulator	Inhibited	-2.4	2.21E-02	metal binding	0.55
4	NM_001042483	**NUPR1**	1.92E-03	5.2	transcription regulator	Inhibited	-7.2	3.27E-09	Cell Cycle	6.49
5	NM_001291738	**CD24**	5.10E-03	5.0	other	Activated	4.4	7.63E-03	chromosome	1.10
6	NM_015832	**MBD2**	4.93E-02	4.5	transcription regulator	Activated	2.8	3.67E-02	N.A.	N.A.
7	NM_005037	**PPARG**	8.29E-04	4.1	ligand-dependent nuclear receptor	Activated	2.0	1.00E+00	Extracellular Region	3.21
8	NM_003707	**RUVBL1**	2.62E-05	2.9	transcription regulator	Activated	2.8	1.63E-03	Histone,Methylation, DNA-Protein Binding	1.95
9	NM_001173989	**RABL6**	5.96E-03	2.8	other	Activated	5.2	5.82E-07	Nuclear Lumen	13.03
10	NM_006618	**KDM5B**	1.10E-03	2.7	transcription regulator	Inhibited	-3.0	1.73E-04	Cell Cycle	4.32
11	NM_001135044	**MAPK9**	1.08E-02	2.5	kinase	Activated	3.0	8.96E-03	N.A.	N.A.
12	NM_001278276	**E2F6**	7.56E-03	2.4	transcription regulator	Inhibited	-3.2	2.88E-07	DNA replication	10.41
13	NM_181659	**NCOA3**	3.07E-03	2.3	transcription regulator	Activated	2.2	1.71E-02	N.A.	N.A.
14	NM_005526	**HSF1**	9.02E-04	2.2	transcription regulator	Inhibited	-2.5	1.24E-04	stress response	2.74
15	NM_012333	**MYCBP**	4.57E-04	2.1	transcription regulator	Activated	2.0	3.66E-02	N.A.	N.A.
16	NM_001206993	**NR1H4**	5.33E-05	-2.2	ligand-dependent nuclear receptor	Inhibited	-2.3	1.23E-01	Xenobiotic Metabolism	2.58
17	NM_000014	**A2M**	5.09E-04	-2.3	transporter	Activated	2.1	1.52E-02	Regulation of Apoptosis	2.64
18	NM_024773	**KDM8**	1.13E-03	-2.7	other	Activated	2.4	4.34E-04	N.A.	N.A.
19	NM_001243514	**ESRRG**	3.26E-02	-3.2	ligand-dependent nuclear receptor	Activated	2.6	2.09E-02	N.A.	N.A.
20	NM_199168	**CXCL12**	9.73E-05	-6.0	cytokine	Inhibited	-2.8	2.85E-01	mutagensis site	1.75

B TP73 up-regulated in tumor (T) compared to non-tumor (NT) tissues.

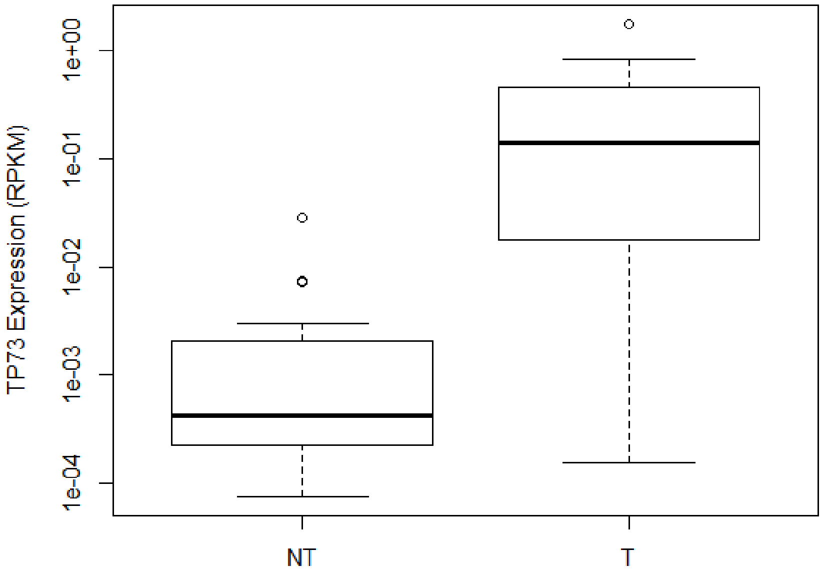

Figure S3. Characteristics of somatic mutations in 25 HCC tumors

A Number of somatic mutations in each patient

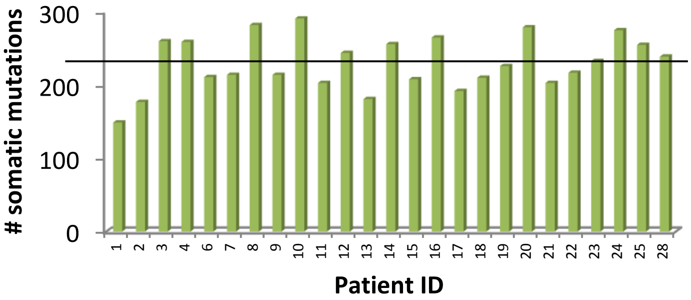

B Somatic mutations that affect start/stop codon, splice donor/acceptor

Functional effect	Gene Name	rs number	cDNA change	Predicted Protein change
Loss of start codon	KAT5		c.2T>C	Loss of start codon which may lead to no protein produced
	UPP1		c.3G>T	
	ZFP62	rs705441	c.3G>C	
Loss of stop codon	LRRC57		c.720A>G	Stop codon mutated to W (*240W) which may lead to additional amino acids after stop codon
	TMEM209		c.1684T>A	Stop codon mutated to K (*562K) which may lead to additional amino acids after stop codon
Splice donor	AMBP		c.117+2T>A	
	APOA2	rs112346700	c.52+2T>C	
	ATP5J2-PTCD1		c.13+1G>T	
	HP		c.265+2T>C	
	SSR4		n.412+1G>C	
	TMEM176B		c.-6+2T>G	
			c.-6+2G>C	
	TXN		c.189+2T>G	
	TXNRD2		c.*65+2T>C	
Splice acceptor	BAHD1		n.356-1G>C	
	MFSD2A		c.94-2A>T	
	MYO18A		c.2039-2A>G	
	USP34		c.10034-2A>T	

C Pathways enriched with deleterious mutations

Category	Term	# genes	Fold Enrichment	FDR
Biological Process	Regulation of small GTPase mediated signal transduction	33	3.11	5.64E-05
	Regulation of hydrolase activity	34	2.39	3.50E-03
	Response to DNA damage stimulus	34	2.16	1.61E-02
	Protein localization	62	1.67	1.95E-02
	Vesicle-mediated transport	45	1.85	2.08E-02
	Cellular response to stress	44	1.84	2.59E-02
	Chromosome organization	39	1.91	3.16E-02
Cellular Compartment	Non-membrane-bounded organelle	151	1.48	4.72E-05
	Extrinsic to membrane	43	2.22	2.08E-04
	Cytosol	86	1.65	2.32E-04
	Membrane-enclosed lumen	112	1.54	2.61E-04
	Cytoskeleton	87	1.61	4.18E-04
	Golgi apparatus	58	1.7	4.28E-03
	Nucleolus	48	1.75	8.61E-03
	Nucleoplasm	56	1.62	1.58E-02
Molecular Function	ATP binding	129	1.99	2.19E-12
	ATPase activity	41	2.8	5.94E-07
	Helicase activity	24	3.9	3.08E-06
	GTPase regulator activity	44	2.48	4.42E-06
	Cytoskeletal protein binding	42	1.9	4.06E-03
	Protein kinase activity	48	1.8	4.26E-03
	Phospholipid binding	21	2.7	4.42E-03
	Enzyme activator activity	31	2.11	6.42E-03
	ATP-dependent helicase activity	14	3.25	1.18E-02
	Cholesterol transporter activity	5	9.49	3.72E-02

Figure S4. Differential gene expression of selected genes in selected HCC patients with NMD mutations

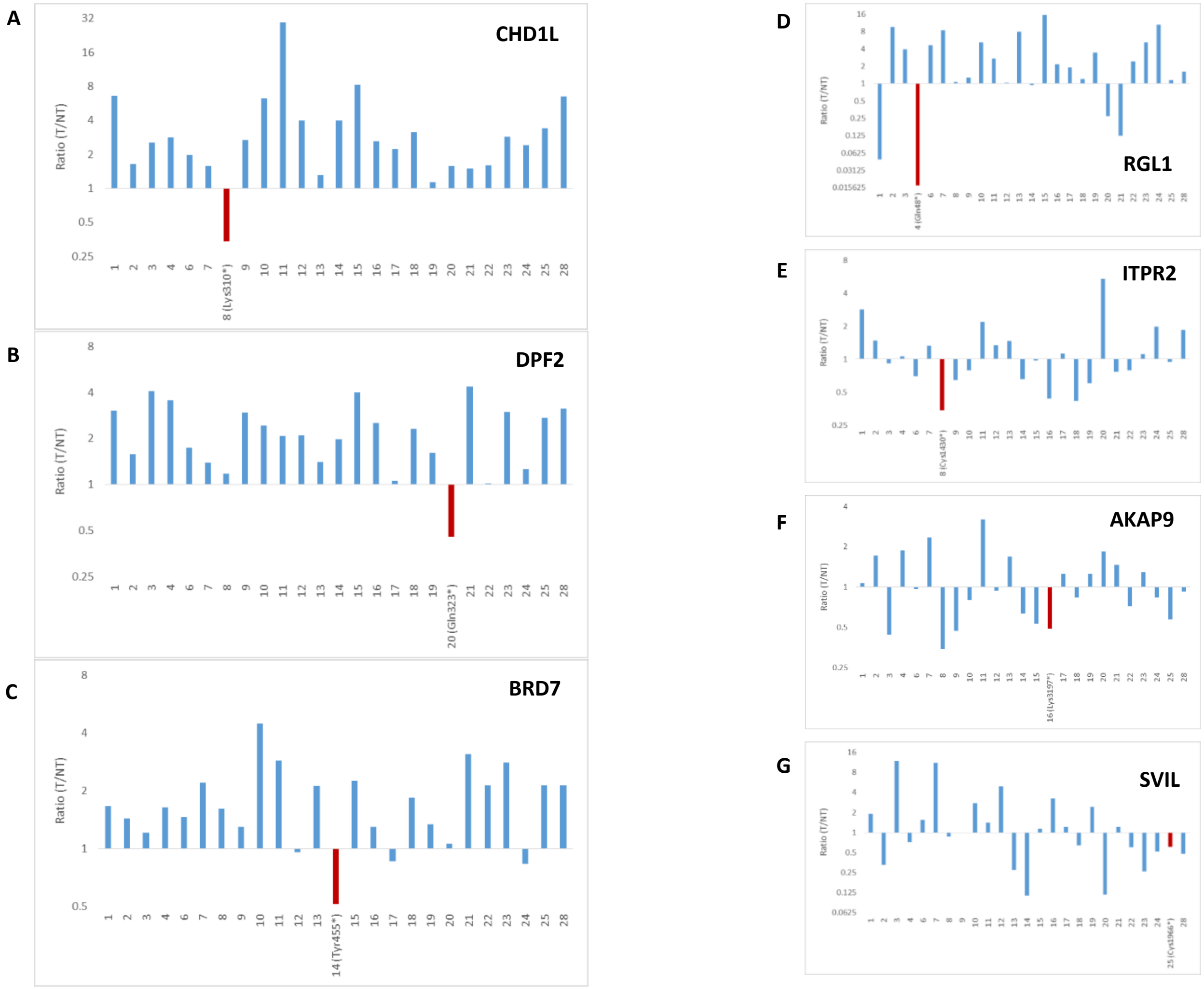

(A) Decreased CHD1L expression was observed in the tumor of patient 8 carrying Lys310* mutation in CHD1L. **(B)** Decreased DPF2 expression was observed in the tumor of patient 20 carrying Gln323* mutation in DPF2. **(C)** Decreased BRD7 expression was observed in the tumor of patient 14 carrying Tyr455* mutation in BRD7. **(D)** Decreased RGL1 expression was observed in the tumor of patient 4 carrying Gln48* mutation in RGL1. **(E)** Decreased ITPR2 expression was observed in the tumor of patient 8 carrying Cys1430* mutation in ITPR2. **(F)** Decreased AKAP9 expression was observed in the tumor of patient 16 carrying Lys3197* mutation in AKAP9. **(G)** Decreased SVIL expression was observed in the tumor of patient 25 carrying Cys1966* mutation in SVIL.

Figure S5. Pathways associated with recurrent mutations.

Category	Term	# genes	Fold Enrichment	FDR
Biological Process	Translation	13	4.18	**3.59E-02**
	Cellular protein localization	14	3.63	**4.64E-02**
	Mitochondrial membrane organization	5	16.64	**4.97E-02**
Cellular Compartment	Ribosome	12	5.62	**1.20E-03**
	Intracellular non-membrane-bounded organelle	48	1.86	**2.00E-03**
	Centrosome	9	4.04	**4.48E-02**
	Cytosol	25	1.89	5.92E-02
	Microtubule cytoskeleton	14	2.57	6.51E-02
Molecular Function	Structural constituent of ribosome	11	6.91	**1.41E-03**
KEGG pathway	Ribosome	10	10.44	**2.24E-05**

Figure S6. Pathways enriched in frequently mutated genes in HCC patients

Category	Term	# mutated genes	# mutations	# patients	Genes with recurrent mutations	Fold enrichment	FDR
Cancer-related	Small cell lung cancer	29	48	20	TP53	1.92	1.72E-02
	Colorectal cancer	24	37	20	CTNNB1, TP53	1.59	1.86E-01
	Pathways in cancer	71	106	25	BID, CTNNB1, TP53	1.21	3.65E-01
	Pancreatic cancer	19	31	20	TP53	1.47	4.20E-01
Signaling	Phosphatidylinositol signaling system	26	44	22	PIP4K2B, SYNJ2	1.96	**2.27E-02**
	Neurotrophin signaling pathway	25	47	23	MAP3K5, TP53	1.12	7.28E-01
	Wnt signaling pathway	29	43	22	CTNNB1, TP53	1.07	7.67E-01
	MAPK signaling pathway	50	76	23	DDIT3, MAP3K5, STK3, TAOK2, TP53	1.04	7.68E-01
	Calcium signaling pathway	26	35	20		0.82	9.76E-01
Cellular Process	Focal adhesion	64	84	25	CTNNB1	1.78	**1.65E-04**
	Endocytosis	60	87	25	DNM1L, HLA-C, NEDD4, PIP4K2B, RAB11FIP5	1.82	**3.10E-04**
	Ubiquitin mediated proteolysis	45	62	24	HERC2, NEDD4, UBE3C	1.83	**2.13E-03**
	Cell cycle	28	43	25	MCM3, TP53	1.25	5.48E-01
Metabolism	Purine metabolism	43	58	23	PNPT1	1.57	5.40E-02
	Inositol phosphate metabolism	19	35	20	PIP4K2B, SYNJ2	1.96	8.40E-02
Miscellaneous	ECM-receptor interaction	31	49	22		2.06	**3.30E-03**
	Tight junction	37	49	23	CTNNB1, MYH14	1.54	9.98E-02
	Ribosome	25	39	21	RPL15, RPL18, RPL18A, RPL27A, RPL8, RPL9, RPLP2, RPS12, RPS24, RPS27	1.6	1.79E-01
	ABC transporters	15	27	20	ABCA2	1.9	1.86E-01
	Progesterone-mediated oocyte maturation	23	32	21		1.49	3.41E-01
	Lysosome	29	47	22	ABCA2, GGA3, NAGPA, SGSH	1.38	3.64E-01
	Leukocyte transendothelial migration	28	36	21	CTNNB1	1.32	4.56E-01
	Regulation of actin cytoskeleton	47	67	23	MYH14, PIP4K2B	1.22	4.64E-01
	Oocyte meiosis	26	33	22		1.32	4.65E-01
	Natural killer cell mediated cytotoxicity	25	38	20	BID, HLA-C, MICA	1.05	8.04E-01
	Huntington's disease	26	46	24	HTT, NDUFB2, TP53	0.81	9.80E-01
	Cytokine-cytokine receptor interaction	30	36	20	RTEL1	0.64	1.00E+00

Figure S7. Human Genes previously reported to be integrated by HBV

Host Gene	Toh et al	Sung et al	Jiang et al	Ding et al	Lau et al	Bonilla Guerrero et al	Zhao et al	Citation Count
TERT	6	28	0	8	1	5	176	6
FN1	0	5	2	5	0	0	21	4
CPS1	0	1	0	1	0	0	2	3
ATRNL1	1	1	0	0	0	0	1	3
ALB	0	0	0	1	0	0	1	2
GRM7	0	1	0	0	0	0	1	2
NRXN1	0	0	0	1	0	0	2	2
RABGAP1L	0	1	0	0	0	0	1	2
ACACA	0	0	0	0	0	0	2	1
ADCY2	0	0	0	0	0	0	1	1
DMD	0	1	0	0	0	0	0	1
GLS2	0	2	0	0	0	0	0	1
GPHN	0	0	0	0	0	0	1	1
HIVEP3	0	0	0	0	0	0	1	1
NR3C2	0	0	0	0	0	0	1	1
NTM	0	0	0	0	0	0	1	1
PRIM2	0	0	0	0	0	0	3	1
SLC26A5	2	0	0	0	0	0	0	1
SLC2A13	0	0	0	1	0	0	0	1
AIP	1	0	0	0	0	0	0	1
FAS	1	0	0	0	0	0	0	1
KMT2B	0	0	0	0	0	0	61	1
KRT32	1	0	0	0	0	0	0	1
PHACTR4	0	0	0	2	0	0	0	1
WWOX	0	0	0	0	0	0	2	1

Figure S8. Summary of sequencing data for 25 HCC patients.

A

**Average number of clean reads**		56,547,541
**Average number of clean bases (bases)**		5,089,278,683
**Average Q20 percentage**		96.76%
**% of reads mapped**	**hg19**	99.97%
	**HBV**	0.03%

B

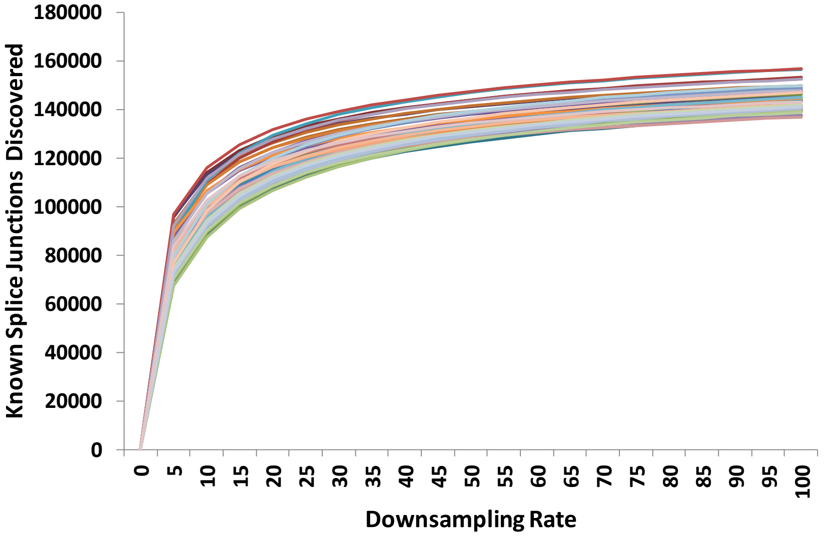

Table S1. Genes significantly associated with clinical characteristics.

				Pathway								Clinical Characteristics					
no	Gene	Fold change (T/N)	p-value	Cell Death and Survial	Cell Cycle	DNA Replication	Cell Assembly	Cell Maintainence	Cell Morphology	Gene Expression	RNA PTM	Edmonson Grade (Poor/Good)	p-value	Vascular Invasion (Yes/No)	P-Value	Survival hazard ratio	P-Value
1	AFP	5.9	2.3E-02									64.09	2.3E-02				
2	ARID3A	4.4	1.5E-04									7.67	1.5E-04				
3	KIAA1524	6.5	2.4E-06									5.10	2.4E-06				
4	KIF11	8.1	2.7E-08									4.09	2.7E-08				
5	LMNB1	4.8	3.5E-07									3.99	3.5E-07				
6	ATAD2	4.3	5.2E-07									3.70	5.2E-07				
7	CBY1	2.0	1.8E-03									3.67	1.8E-03				
8	MID1IP1	4.3	3.0E-07									3.58	3.0E-07				
9	CENPJ	2.4	7.1E-04									3.51	7.1E-04				
10	TPD52L2	3.1	1.2E-05									3.46	1.2E-05				
11	ARHGAP19	2.4	5.3E-05									3.40	5.3E-05				
12	WHSC1	3.4	1.2E-06									3.37	1.2E-06				
13	BTBD3	2.2	7.5E-04									3.23	7.5E-04				
14	TTLL4	2.7	2.2E-05									3.20	2.2E-05				
15	RPSAP58	2.6	5.9E-05									3.06	5.9E-05				
16	ARL6IP6	2.3	8.3E-05									3.03	8.3E-05				
17	SEC23B	3.0	4.7E-06									2.93	4.7E-06				
18	STK35	2.4	1.2E-05									2.92	1.2E-05				
19	CPNE1	2.6	2.1E-05									2.87	2.1E-05				
20	NCAPD2	3.9	3.1E-07									2.83	3.1E-07				
21	ABHD3	2.1	6.1E-05									2.83	6.1E-05				
22	KIF2A	3.1	3.7E-06									2.80	3.7E-06				
23	GPR107	2.3	5.3E-05									2.79	5.3E-05				
24	CPD	2.9	1.4E-06									2.78	1.4E-06				
25	VPS54	2.0	1.1E-04									2.62	1.1E-04				
26	LBR	2.1	7.5E-06									2.52	7.5E-06				
27	ANKRD52	3.5	5.5E-08									2.49	5.5E-08				
28	STRBP	2.4	8.7E-07									2.45	8.7E-07				
29	POLA1	2.9	8.3E-07									2.45	8.3E-07				
30	TFAP4	2.5	1.6E-06									2.43	1.6E-06				
31	HNRNPC	2.4	2.0E-06									2.42	2.0E-06				
32	POU2F1	2.5	1.6E-06									2.40	1.6E-06				
33	ACD	2.1	1.6E-05									2.34	1.6E-05				
34	HIF1AN	2.0	5.4E-05									2.32	5.4E-05				
35	FNDC3B	2.1	3.1E-06									2.32	3.1E-06				
36	TOPBP1	2.8	2.4E-07									2.30	2.4E-07				
37	TRIP12	2.0	2.2E-05									2.29	2.2E-05				
38	ZBED4	2.3	1.7E-06									2.23	1.7E-06				
39	FAM168B	2.0	5.6E-06									2.13	5.6E-06				
40	QSER1	2.1	2.2E-06									2.09	2.2E-06				
41	IKBKAP	2.1	1.6E-06									2.01	1.6E-06				
42	TULP3	2.4	4.7E-07									2.00	4.7E-07				
43	U2SURP	2.2	1.2E-06									1.96	1.2E-06				
44	B4GALT3	2.6	1.0E-07									1.96	1.0E-07				
45	MIB1	2.0	1.4E-06									1.90	1.4E-06				
46	KHSRP	2.2	4.1E-07									1.90	4.1E-07				
47	MAPK1	2.1	2.3E-07									1.85	2.3E-07				
48	ANAPC1	2.3	6.8E-08									1.84	6.8E-08				
49	ARID4B	2.1	2.2E-07									1.79	2.2E-07				
50	NCSTN	2.3	7.6E-08									1.75	7.6E-08				
51	SKP2	3.0	1.0E-05									3.24	1.0E-05			13.86	2.4E-02
52	TBL1X	2.0	1.3E-03									3.80	1.3E-03			7.39	2.4E-02
53	NUP133	2.4	2.5E-08									1.73	2.5E-08			79.61	4.7E-02
54	MGST1	-2.4	2.0E-04									-3.66	2.0E-04				
55	SLC46A3	-2.4	3.2E-03									-4.86	3.2E-03				
56	NR1H4	-2.5	1.6E-03									-4.93	1.6E-03				
57	APOC3	-2.2	7.3E-03									-4.98	7.3E-03				
58	ASPDH	-4.5	3.7E-05									-5.69	3.7E-05				
59	CYP2A6	-11.2	4.7E-04									-39.13	4.7E-04				
60	AKR7A3	-4.7	1.3E-04									-7.17	1.3E-04			0.22	3.3E-02
61	SHMT1	-2.1	9.0E-03									-5.54	9.0E-03			0.12	4.65
62	AP2A1	2.01	2.2E-07													1849.70	1.6E-02
63	SLC30A6	2.44	1.7E-07													349.14	1.1E-02
64	ERCC2	2.35	9.3E-08													179.04	1.6E-02
65	NSL1	2.24	1.1E-08													91.27	2.3E-02

66	QPCTL	2.36	7.2E-07						**58.48**	**4.6E-02**
67	PDZK1	3.37	1.7E-07						**44.10**	**3.5E-02**
68	POLR2H	2.04	3.2E-04						**39.99**	**3.7E-02**
69	RAD17	2.21	8.7E-05						**39.85**	**1.5E-02**
70	ADAR	2.28	6.6E-08						**33.36**	**4.6E-02**
71	ADNP	2.34	1.2E-06						**31.98**	**3.0E-02**
72	ZNF765	2.39	1.3E-06						**30.86**	**3.7E-02**
73	ZNF552	2.34	1.3E-05						**30.32**	**2.9E-02**
74	SLMAP	2.12	8.0E-05						**27.20**	**4.3E-02**
75	RFWD2	2.56	2.8E-07						**24.27**	**4.5E-02**
76	RSRC1	2.69	5.4E-05						**24.09**	**1.0E-02**
77	RPS6KA3	2.33	2.0E-05						**23.47**	**2.1E-02**
78	ZNF585A	2.30	1.7E-05						**21.17**	**3.6E-02**
79	FAM63A	2.01	8.0E-05						**16.58**	**3.3E-02**
80	WDR5	2.32	4.3E-05						**15.99**	**4.9E-02**
81	LOC730202	2.40	2.0E-04						**13.86**	**3.0E-02**
82	DESI2	2.40	8.5E-06						**12.89**	**2.5E-02**
83	UCHL5	3.27	6.9E-06						**11.94**	**2.0E-02**
84	FAT1	2.63	1.4E-05						**11.64**	**5.0E-02**
85	MBNL3	2.32	1.4E-03						**7.84**	**1.5E-02**
86	YIF1B	2.59	1.3E-03						**7.52**	**3.0E-02**
87	YY1AP1	4.57	1.2E-07						**7.00**	**2.9E-02**
88	EIF2B4	2.15	6.1E-04						**6.17**	**5.0E-02**
89	SAA4	-4.22	3.9E-04						**0.31**	**2.9E-02**
90	CDA	-2.51	9.3E-03						**0.27**	**1.6E-02**
91	SERPINB8	-2.09	1.1E-02						**0.24**	**4.9E-02**
92	TMEM45A	-3.66	1.6E-04						**0.24**	**2.4E-02**
93	CYP2B7P	-4.54	2.2E-03						**0.20**	**3.5E-02**
94	COLEC11	-2.35	2.4E-02						**0.19**	**2.7E-02**
95	FXYD1	-2.18	2.6E-02						**0.19**	**1.7E-02**
96	ABCA8	-2.38	5.0E-03						**0.19**	**4.7E-02**
97	AKR1D1	-3.58	2.8E-04						**0.16**	**4.6E-02**
98	MCC	-2.04	4.4E-03						**0.15**	**2.3E-02**
99	IYD	-3.34	1.0E-04						**0.15**	**2.9E-02**
100	AZGP1P1	-2.95	5.9E-04						**0.14**	**1.3E-02**
101	PAIP2B	-2.16	6.6E-03						**0.13**	**1.3E-02**
102	GCH1	-2.20	3.0E-04						**0.10**	**3.0E-02**
103	AKR7L	-2.27	8.2E-04						**0.09**	**2.3E-02**
104	LOC101927755	-2.01	4.5E-03						**0.05**	**6.6E-03**
105	SLC27A2	-2.07	1.9E-04						**0.05**	**2.1E-02**
106	C14orf105	-2.12	8.0E-03						**0.04**	**1.7E-02**
107	NME1-NME2	2.0	3.3E-03				**4.47**	**3.3E-03**		
108	ZNF623	2.6	1.1E-05				**2.94**	**1.1E-05**		
109	APAF1	2.1	2.3E-04				**2.72**	**2.3E-04**		
110	TCERG1	2.1	7.2E-06				**2.26**	**7.2E-06**		

Table S2. Characteristics of the HBV-Human Chimeric Transcripts

Host Gene	Region	HBV Gene	Location	# Chimeric Transcripts	Regulatory elements affected	Details
TERT	promoter	X,PreC	Tumor	3	TFBS	+VDR, CAR, PXR|+VDR|-E2F|-Pax-5|-ZF5
TERT	promoter	X	Tumor	3	TFBS	+CP2/LBP-1c/LSF|+Pax-4|+SREBP|+SREBP-1|+ZF5|-Zic3
TERT	promoter	X	Tumor	3	TFBS	+Osf2|+myogenin / NF-1|+GR|+HNF4|+HNF4alpha|-Hand1:E47
GATA3	non-coding exon	C	Tumor	2		
GATA3	non-coding exon	C, P	Tumor	2	ESE/ESS	+FB_ESE_151|+HF_ESE_791
DTNA	intron	X	Tumor	2		
DTNA	intron	S, P	Tumor	2		
SCO1	intron	P	Tumor	2	ISRE	-GY_DS_ISRE_14
SCO1	intron	C, P	Tumor	2		
FN1	intron	S, P	Non-Tumor	2		
FN1	intron	S, P	Non-Tumor	2	ISRE	-GY_DS_ISRE_30
PAK2	intron	X	Non-Tumor	2		
PAK2	intron	X	Non-Tumor	2		
MUC20	promoter	X	Non-Tumor	1	TFBS	+MYB|+Nkx2-5|+Pax-2|-myogenin / NF-1|-NF-1|-PPARgamma:RXRalpha, PPARgamma|-Pax-5|-Pax-6|-SREBP
SCAI	promoter	X	Non-Tumor	1	TFBS	+BRCA1:USF2|+FAC1|-CDP|-Hand1:E47|-FOXJ2
GLS2	promoter	X,PreC	Non-Tumor	1	TFBS	+FOXJ2|+Nkx2-5|+Pax-6|+Pax-8|+RUSH-1alpha|+SREBP|+TBP|-Ebox|-TFE|-USF2|-GATA-X|-MEIS1B:HOXA9|VDR, CAR, PXR|-Oct-01
IFT52	promoter	X,PreC	Non-Tumor	1	TFBS	+FOXJ2|+Oct-4 (POU5F1)|-p300|-Pax-5|-RUSH-1alpha
DHX9	intron	C	Non-Tumor	1	ISRE	+GY_DS_ISRE_147
ADCY2	intron	X,PreC	Non-Tumor	1		
CALD1	intron	X,PreC	Non-Tumor	1		
COL25A1	intron	X,PreC	Non-Tumor	1		
CPS1	intron	X,PreC	Non-Tumor	1		
CTNNA2	intron	X,PreC	Non-Tumor	1		
DAB1	intron	X,PreC	Non-Tumor	1		
DENND4C	intron	X,PreC	Non-Tumor	1		
FAM189A1	intron	X,PreC	Non-Tumor	1		
FBXO28	intron	X,PreC	Non-Tumor	1		
FCHSD2	intron	X,PreC	Non-Tumor	1		
GPD2	intron	X,PreC	Non-Tumor	1		
GPHN	intron	X,PreC	Non-Tumor	1		
GRM7	intron	X,PreC	Non-Tumor	1		
IL15RA	intron	X,PreC	Non-Tumor	1		
KLHL7	intron	X,PreC	Non-Tumor	1	ISRE	-GY_DS_ISRE_136
MAP2K5	intron	X,PreC	Non-Tumor	1		
MYLK	intron	X,PreC	Non-Tumor	1	ISRE	+GY_DS_ISRE_21|-GY_DS_ISRE_71|-GY_DS_ISRE_133|-GY_DS_ISRE_136
NRXN1	intron	X,PreC	Non-Tumor	1		
NTM	intron	X,PreC	Non-Tumor	1		
PDGFD	intron	X,PreC	Non-Tumor	1		
PRKD2	intron	X,PreC	Non-Tumor	1		
RNF180	intron	X,PreC	Non-Tumor	1		
SMAP2	intron	X,PreC	Non-Tumor	1		
TJP1	intron	X,PreC	Non-Tumor	1		
VAT1L	intron	X,PreC	Non-Tumor	1		
WDR19	intron	X,PreC	Non-Tumor	1		
MICU1	intron	S, P	Non-Tumor	1	ISRE	+GY_DS_ISRE_128
CNGA1	intron	X, P	Non-Tumor	1		
ANXA1	intron	X, P	Non-Tumor	1		
ACACA	intron	X	Non-Tumor	1		
ADAMTS6	intron	X	Non-Tumor	1		
ADNP2	intron	X	Non-Tumor	1		
ALS2CR11	intron	X	Non-Tumor	1		
ANKH	intron	X	Non-Tumor	1		
AVIL	intron	X	Non-Tumor	1		
C12orf42	intron	X	Non-Tumor	1		
FMR1	intron	X	Non-Tumor	1	ISRE	-GY_DS_ISRE_23|-GY_DS_ISRE_28
NOLC1	intron	X	Non-Tumor	1	ISRE	+GY_DS_ISRE_158
CACYBP	intron	X	Non-Tumor	1	ISRE	-GY_DS_ISRE_21|-GY_DS_ISRE_60
CDC45	intron	X	Non-Tumor	1		
CNP	intron	X	Non-Tumor	1		
CYFIP2	intron	X	Non-Tumor	1		
DMBT1	intron	X	Non-Tumor	1		
DMD	intron	X	Non-Tumor	1		
DOCK1	intron	X	Non-Tumor	1		
DPP4	intron	X	Non-Tumor	1		
EFHC2	intron	X	Non-Tumor	1		
FLRT2	intron	X	Non-Tumor	1		
FMNL2	intron	X	Non-Tumor	1		
GALNT2	intron	X	Non-Tumor	1		
HIVEP3	intron	X	Non-Tumor	1		
KHDRBS2	intron	X	Non-Tumor	1		
LOC101928135	intron	X	Non-Tumor	1		
MACROD2	intron	X	Non-Tumor	1		
NFKBID	intron	X	Non-Tumor	1		
NR3C2	intron	X	Non-Tumor	1		
OSMR-AS1	intron	X	Non-Tumor	1		

OTUD7A	intron	X	**Non-Tumor**	**1**		
PDE8A	intron	X	**Non-Tumor**	**1**		
PLCH1	intron	X	**Non-Tumor**	**1**		
PRIM2	intron	X	**Non-Tumor**	**1**		
RABGAP1L	intron	X	**Non-Tumor**	**1**		
SIK2	intron	X	**Non-Tumor**	**1**		
RALGPS2	intron	X	**Non-Tumor**	**1**		
RASSF1	5'UTR	X	**Non-Tumor**	**1**		
SLC17A2	5'UTR	X	**Non-Tumor**	**1**	ESE/ESS	+FB_ESE_138|+FB_ESE_184|-FB_ESE_63|-HF_ESE_895
SLC26A5	intron	X	**Non-Tumor**	**1**	ISRE	-GY_DS_ISRE_78
SLC2A13	intron	C	**Non-Tumor**	**1**		
SLCO1A2	intron	X	**Non-Tumor**	**1**		
TMEM63A	intron	X	**Non-Tumor**	**1**		
TMEM65	intron	X	**Non-Tumor**	**1**		
WDR90	intron	X	**Non-Tumor**	**1**		
XPR1	intron	X	**Non-Tumor**	**1**		
GTF2IP1	non-coding exon	X	**Non-Tumor**	**1**	ESE/ESS	+FB_ESE_161|+HF_ESE_410|-HF_ESE_412
ALB	coding exon	P	**Non-Tumor**	**1**	ESE/ESS	-HF_ESE_1085
AHCYL1	intron	C	**Tumor**	**1**		
ADPRM	intron	C, P	**Tumor**	**1**	ISRE	-GY_DS_ISRE_72|-GY_DS_ISRE_91|-GY_DS_ISRE_148
KMT2B	intron	X, P	**Tumor**	**1**		
DISP1	intron	X,PreC	**Tumor**	**1**		
PARP6	intron	X,PreC	**Tumor**	**1**	ISRE	+GY_DS_ISRE_76|+GY_DS_ISRE_84|-GY_DS_ISRE_112
TGM2	intron	X,PreC	**Tumor**	**1**		
WWOX	intron	X,PreC	**Tumor**	**1**		
ZC3H3	intron	X,PreC	**Tumor**	**1**		
AIP	intron	X	**Tumor**	**1**		
ATRNL1	intron	X	**Tumor**	**1**		
ATRNL1	intron	X	**Tumor**	**1**		
DDX3X	intron	X	**Tumor**	**1**		
EEF2KMT	intron	X	**Tumor**	**1**		
MARCH8	intron	X	**Tumor**	**1**		
RAPGEF5	intron	X	**Tumor**	**1**		
FAS	intron	X	**Tumor**	**1**	ISRE	+GY_DS_ISRE_1|-GY_DS_ISRE_86
KRT32	coding exon	X	**Tumor**	**1**	ESE/ESS	+SC35_2|+HF_ESE_235
PHACTR4	3'UTR	C	**Tumor**	**1**	ESE/ESS	+HF_ESS_374|-HF_ESS_427
SON	3'UTR	P, S	**Tumor**	**1**	ESE/ESS	+HF_ESE_1243|-FB_ESE_213
GATA3-AS1	non-coding exon	C, P	**Tumor**	**1**		

Table S3. Comparison of genes associated with clinical characteristics identified in this study and TCGA project.

		Histological grade				Vascular invasion				Overall survival			
		This study (n=25)		TCGA (n=366)		This study (n=25)		TCGA (n=315)		This study (n=25)		TCGA (n=371)	
No.	Gene	Fold change (Poor/Good)	P-value	Regression Coefficient	p-value	Vascular Invasion (Yes/No)	P-value	Regression Coefficient	P-value	Hazard Ratio	P-Value	Survival hazard ratio	P-Value
1	AFP	64.1	2.3E-02	0.14	2.9E-05								
2	ARID3A	7.67	1.5E-04	0.43	1.2E-05								
3	KIAA1524	5.10	2.4E-06	0.91	2.1E-06								
4	KIF11	4.09	2.7E-08	0.79	3.3E-08								
5	LMNB1	3.99	3.5E-07	0.62	5.1E-10								
6	ATAD2	3.70	5.2E-07	0.61	3.4E-08								
7	CBY1	3.67	1.8E-03	1.02	1.4E-06								
8	MID1IP1	3.58	3.0E-07	0.33	1.5E-03								
9	CENPJ	3.51	7.1E-04	1.33	3.1E-08								
10	TPD52L2	3.46	1.2E-05	1.10	8.7E-11								
11	ARHGAP19	3.40	5.3E-05	1.00	8.7E-05								
12	BTBD3	3.23	7.5E-04	0.56	2.5E-04								
13	TTLL4	3.20	2.2E-05	0.66	8.2E-08								
14	ARL6IP6	3.03	8.3E-05	0.62	2.7E-04								
15	STK35	2.92	1.2E-05	0.56	1.8E-03								
16	CPNE1	2.87	2.1E-05	0.57	1.7E-05								
17	NCAPD2	2.83	3.1E-07	0.66	1.9E-07								
18	ABHD3	2.83	6.1E-05	0.40	8.3E-04								
19	KIF2A	2.80	3.7E-06	0.64	4.5E-04								
20	GPR107	2.79	5.3E-05	0.65	1.4E-04								
21	CPD	2.78	1.4E-06	0.33	2.4E-03								
22	VPS54	2.62	1.1E-04	0.91	4.7E-07								
23	LBR	2.52	7.5E-06	0.84	9.7E-10								
24	ANKRD52	2.49	5.5E-08	0.69	1.1E-05								
25	STRBP	2.45	8.7E-07	1.09	1.6E-06								
26	POLA1	2.45	8.3E-07	0.90	5.3E-06								
27	TFAP4	2.43	1.6E-06	1.40	9.6E-07								
28	HNRNPC	2.42	2.0E-06	1.19	2.8E-07								
29	POU2F1	2.40	1.6E-06	1.63	5.3E-07								
30	ACD	2.34	1.6E-05	0.82	2.9E-05								
31	HIF1AN	2.32	5.4E-05	0.61	9.4E-03								
32	FNDC3B	2.32	3.1E-06	0.58	6.6E-05								

33	TOPBP1	2.30	2.4E-07	0.66	3.3E-05								
34	ZBED4	2.23	1.7E-06	0.72	2.6E-04								
35	FAM168B	2.13	5.6E-06	0.33	2.3E-02								
36	QSER1	2.09	2.2E-06	0.77	3.4E-04								
37	TULP3	2.00	4.7E-07	0.83	1.3E-06								
38	U2SURP	1.96	1.2E-06	0.97	1.8E-07								
39	B4GALT3	1.96	1.0E-07	0.82	3.2E-06								
40	MIB1	1.90	1.4E-06	0.32	3.8E-02								
41	KHSRP	1.90	4.1E-07	0.56	5.6E-04								
42	MAPK1	1.85	2.3E-07	0.41	1.2E-02								
43	ANAPC1	1.84	6.8E-08	1.33	9.5E-06								
44	ARID4B	1.79	2.2E-07	1.04	1.1E-06								
45	NCSTN	1.75	7.6E-08	0.38	7.2E-03								
46	SKP2	3.24	1.0E-05	0.47	6.0E-05					13.86	2.4E-02	1.44	3.5E-04
47	NUP133	1.73	2.5E-08	0.67	4.4E-04					79.61	4.7E-02	1.34	8.0E-02
48	SEC23B	2.93	4.7E-06	0.23	1.2E-01								
49	TRIP12	2.29	2.2E-05	0.31	1.3E-01								
50	MGST1	-3.66	2.0E-04	-0.20	1.1E-02								
51	SLC46A3	-4.86	3.2E-03	-0.39	1.7E-07								
52	APOC3	-4.98	7.3E-03	-0.17	2.5E-04								
53	ASPDH	-5.69	3.7E-05	-0.32	8.9E-08								
54	CYP2A6	-39.1	4.7E-04	-0.19	8.0E-10								
55	SHMT1	-5.54	9.0E-03	-0.47	9.9E-08					0.12	4.65	0.85	2.1E-02
56	AKR7A3	-7.17	1.3E-04	-0.20	1.5E-04					0.22	3.3E-02	0.88	5.2E-03
57	NR1H4	-4.93	1.6E-03	-0.22	1.1E-01								
58	WHSC1	3.37	1.2E-06										
59	RPSAP58	3.06	5.9E-05										
60	IKBKAP	2.01	1.6E-06										
61	TBL1X	3.80	1.3E-03	-0.20	2.0E-01					7.39	2.4E-02	1.36	2.1E-02
62	NME1-NME2					4.47	3.3E-03	0.35	1.8E-02				
63	ZNF623					2.94	1.1E-05	0.26	2.3E-01				
64	APAF1					2.72	2.3E-04	0.48	7.4E-02				
65	TCERG1					2.26	7.2E-06	0.39	9.0E-02				
66	ADAR									33.36	4.6E-02	1.41	1.4E-02
67	ADNP									31.98	3.0E-02	1.58	2.2E-03
68	AP2A1									1850	1.6E-02	1.44	3.8E-02
69	DESI2									12.89	2.5E-02	1.36	1.8E-02
70	EIF2B4									6.17	5.0E-02	2.62	1.4E-06
71	ERCC2									179	1.6E-02	1.52	1.1E-02
72	NSL1									91.27	2.3E-02	1.55	1.2E-02
73	POLR2H									39.99	3.7E-02	1.64	2.5E-04
74	RSRC1									24.09	1.0E-02	1.63	1.0E-02
75	SLC30A6									349	1.1E-02	1.91	2.8E-04
76	WDR5									15.99	4.9E-02	1.48	1.4E-02
77	YIF1B									7.52	3.0E-02	1.66	6.1E-05

78	YY1AP1									**7.00**	**2.9E-02**	**1.66**	**1.3E-03**
79	ZNF765									**30.86**	**3.7E-02**	**4.02**	**2.1E-04**
80	ABCA8									**0.19**	**4.7E-02**	**0.80**	**2.5E-02**
81	AKR1D1									**0.16**	**4.6E-02**	**0.88**	**5.2E-03**
82	IYD									**0.15**	**2.9E-02**	**0.69**	**1.1E-03**
83	PAIP2B									**0.13**	**1.3E-02**	**0.80**	**4.2E-02**
84	SAA4									**0.31**	**2.9E-02**	**0.92**	**2.7E-02**
85	FAT1									**11.64**	**5.0E-02**	1.08	3.8E-01
86	PDZK1									**44.10**	**3.5E-02**	1.02	8.2E-01
87	QPCTL									**58.48**	**4.6E-02**	1.23	9.0E-02
88	RAD17									**39.85**	**1.5E-02**	1.57	5.7E-02
89	RFWD2									**24.27**	**4.5E-02**	1.34	6.0E-02
90	RPS6KA3									**23.47**	**2.1E-02**	1.18	1.0E-01
91	SLMAP									**27.20**	**4.3E-02**	1.48	6.4E-02
92	UCHL5									**11.94**	**2.0E-02**	1.31	8.3E-02
93	ZNF552									**30.32**	**2.9E-02**	1.23	2.6E-01
94	ZNF585A									**21.17**	**3.6E-02**	1.88	8.7E-02
95	AKR7L									**0.09**	**2.3E-02**	0.85	1.3E-01
96	COLEC11									**0.19**	**2.7E-02**	0.96	5.2E-01
97	FXYD1									**0.19**	**1.7E-02**	0.93	1.1E-01
98	GCH1									**0.10**	**3.0E-02**	0.93	4.0E-01
99	SLC27A2									**0.05**	**2.1E-02**	0.90	5.9E-02
100	FAM63A									**16.58**	**3.3E-02**		
101	LOC730202									**13.86**	**3.0E-02**		
102	CYP2B7P									**0.20**	**3.5E-02**		
103	AZGP1P1									**0.14**	**1.3E-02**		
104	LOC101927755									**0.05**	**6.6E-03**		
105	MBNL3									**7.84**	**1.5E-02**	0.98	7.6E-01
106	C14orf105									**0.04**	**1.7E-02**	1.20	6.2E-02
107	CDA									**0.27**	**1.6E-02**	1.06	3.4E-01
108	MCC									**0.15**	**2.3E-02**	1.14	3.5E-01
109	SERPINB8									**0.24**	**4.9E-02**	1.14	3.2E-01
110	TMEM45A									**0.24**	**2.4E-02**	**1.18**	**6.3E-03**

Higher expression of genes associated with poor prognosis are hilighed in red (p-value<0.05) and pink (p-value>0.05) while lower expression of genes associated with poor prognosis are higlighted in dark (p-value<0.05) and light (p-value>0.05) green, respectively. P-values<0.05 were labelled as bold.
